# Chronic stress induces pulmonary epithelial cells to produce acetylcholine that remodels lung pre-metastatic niche of breast cancer by enhancing NETosis

**DOI:** 10.1186/s13046-023-02836-5

**Published:** 2023-09-29

**Authors:** Jun Pan, Leyi Zhang, Xiaomei Wang, Lili Li, Chenghui Yang, Zhen Wang, Ke Su, Xiaoxiao Hu, Yi Zhang, Guohong Ren, Jiahuan Jiang, Peng Li, Jian Huang

**Affiliations:** 1 Department of Breast Surgery, Key Laboratory of Tumor Microenvironment and Immune Therapy of Zhejiang Province, Second Affiliated Hospital, Zhejiang University School of Medicine, 88 Jiefang Road, Hangzhou, 310009 P.R. China; 2 Key Laboratory of Tumor Microenvironment and Immune Therapy of Zhejiang Province, Second Affiliated Hospital, Zhejiang University School of Medicine, Hangzhou, 310009 P.R. China; 3 Cancer Institute, Second Affiliated Hospital, Zhejiang University School of Medicine, Hangzhou, 310009 P.R. China; 4 Department of Pathology, Second Affiliated Hospital, Zhejiang University School of Medicine, Hangzhou, 310009 P.R. China; 5 Department of Oncology, Second Affiliated Hospital, Zhejiang University School of Medicine, Hangzhou, 310009 Zhejiang China; 6 Department of Breast Surgery, First Affiliated Hospital of Wenzhou Medical University, Wenzhou, 325000 P.R. China

**Keywords:** Breast cancer, Lung metastasis, Chronic stress, Acetycholine, Neutrophils, NETosis

## Abstract

**Background:**

Chronic stress promotes most hallmarks of cancer through impacting the malignant tissues, their microenvironment, immunity, lymphatic flow, etc. Existing studies mainly focused on the roles of stress-induced activation of systemic sympathetic nervous system and other stress-induced hormones, the organ specificity of chronic stress in shaping the pre-metastatic niche remains largely unknown. This study investigated the role of chronic stress in remodeling lung pre-metastatic niche of breast cancer.

**Methods:**

Breast cancer mouse models with chronic stress were constructed by restraint or unpredictable stress. Expressions of tyrosine hydroxylase, vesicular acetylcholine transporter (VAChT), EpCAM and NETosis were examined by immunofluorescence and confocal microscopy. mRNA and protein levels of choline acetyltransferase (ChAT), VAChT, and peptidylarginine deiminase 4 were detected by qRT-PCR and Western blotting, respectively. Immune cell subsets were analyzed by flow cytometry. Acetylcholine (ACh) and chemokines were detected by ELISA and multi chemokine array, respectively. ChAT in lung tissues from patients was examined by immunohistochemistry.

**Results:**

Breast cancer-bearing mice suffered chronic stress metastasized earlier and showed more severe lung metastasis than did mice in control group. VAChT, ChAT and ChAT^+^ epithelial cells were increased significantly in lung of model mice undergone chronic stress. ACh and chemokines especially CXCL2 in lung culture supernatants from model mice with chronic stress were profoundly increased. Chronic stress remodeled lung immune cell subsets with striking increase of neutrophils, enhanced NETosis in lung and promoted NETotic neutrophils to capture cancer cells. ACh treatment resulted in enhanced NETosis of neutrophils. The expression of ChAT in lung tissues from breast cancer patients with lung metastasis was significantly higher than that in patients with non-tumor pulmonary diseases.

**Conclusions:**

Chronic stress promotes production of CXCL2 that recruits neutrophils into lung, and induces pulmonary epithelial cells to produce ACh that enhances NETosis of neutrophils. Our findings demonstrate for the first time that chronic stress induced epithelial cell derived ACh plays a key role in remodeling lung pre-metastatic niche of breast cancer.

**Supplementary Information:**

The online version contains supplementary material available at 10.1186/s13046-023-02836-5.

## Background

Psychological stress is a well-recognized risk factor that plays a pivotal role in cancer initiation, progression, invasion, metastasis and recurrence [1–4]. Epidemiological studies have demonstrated that patients with cancer tend to suffer chronic psychological stress and these patients often have a worse prognosis [5–7]. Use of anti-depressants and blockage of stress-induced hormones prolong the progression-free survival (PFS) and overall survival (OS) of patients with cancer [8, 9], demonstrating a crucial role of psychological depressor in the treatment of cancer.

Breast cancer is the most common cancer in females worldwide with a dramatic increase in incidence [10, 11]. Due to the rapid development of screening methods, early diagnosis and treatment methods, the mortality rate of breast cancer in women has slowed down since 1989 in the United States [10]. However, it is worth noting that the mortality rate is still on the rise in developing countries [12]. Patients with breast cancer suffer a lot of stressors, such as the destroy of femaleness, abnormal physiological state, side effects of medication, decline in quality of life, leading to the cause of chronic psychological stress. A meta-analysis which contained 282,203 patients with breast cancer demonstrates that breast cancer-specific mortality is correlated with the state of depression, and patients suffered both breast cancer and depression have a worse prognosis [13]. Among various factors to breast cancer-specific mortality, metastasis is the leading cause of death in patients [14].

In recent years, great progress has been made in the mechanisms by which psychological stress promotes metastasis of breast cancer. Chronic stress promotes dissemination of breast cancer cells by remodeling lymph vasculature [15] and epithelial to mesenchymal transition (EMT)-mediated metastasis of breast cancer through activation of STAT3 signaling pathway [16]. Chronic stress-induced activation of β2-adrenoceptor results in elevated cAMP and increased intracellular calcium that enhance expression of vascular endothelial growth factor (VEGF), matrix metalloproteinase 2 (MMP2) and MMP9, hereby enhancing the invasiveness of breast cancer cells [17]. Chronic stress-induced epinephrine promotes breast cancer stem cell-like properties via activation of lactate dehydrogenase A (LDHA) -dependent metabolic rewiring [18]. It was also found that the stress hormone glucocorticoid cortisol promotes metastasis of breast cancer via promoting expression of inducible nitric oxide synthase, angiogenesis and expression of the metastasis-related gene TWIST1 [19]. While, An et al. demonstrated that chronic stress may accumulate myeloid-derived suppressor cells (MDSCs) via activation of β-adrenergic signaling and IL-6/STAT3 pathway, thereby promoting breast carcinoma metastasis [20]. Therefore, existing studies mainly focused on the roles of stress-induced activation of systemic sympathetic nervous system and other stress-induced hormones in the dissemination of breast cancer, the function and organ specificity of chronic stress in shaping the pre-metastatic microenvironment remain largely unknown.

By using multiple mouse breast cancer models, we show, for the first time, that chronic stress promotes pulmonary epithelial cells to secrete acetylcholine (ACh) that remodels the pre-metastatic niche by enhancing neutrophil extracellular trap formation or NETosis.

## Materials and methods

### Cell culture

The 4T1 cell line (ATCC: CRL-2539) was purchased form the Shanghai Institute of Cell Biology of the Chinese Academy of Science (Shanghai, China). Cells were cultured in RPMI 1640 medium (Gibco, Rockville, USA) containing 10% fetal bovine serum (Gibco, Rockville, USA) and 1% penicillin-streptomycin (Gibco, Rockville, USA) at 37℃ in a humidified incubator under 5% CO_2_.

### Murine breast cancer models

To observe the progression of breast cancer metastasis, we established three different breast cancer-bearing mice models. Wild-type BALB/c female mice aged six to eight weeks were purchased from Shanghai SLAC Laboratory Animal Co., Ltd. (Shanghai, China). PyMT-MMTV transgenic mice were a kind gift from Professor Qiyang Shou (Zhejiang Chinese Medical University) and were used as the spontaneous breast cancer model. Before dividing into groups, gene identification was performed to all female mice and only transgenic mice were adopted for subsequent studies. For orthotopic injection model, BALB/c mice were anesthetized with an intraperitoneal injection of 1.0% pentobarbital and then 100 μl of cell suspension (4T1 cells, 1 × 10^6^ cells/ml) were injected into the right fourth mammary fat pad. During the establishment of orthotopic 4T1 model, the largest diameter (LD) and the smallest diameter (SD) of tumors were measured every 3 days. The tumor volume (TV) was calculated according to the previously reported formula: TV (mm^3^) = (LD×SD^2^)×0.52 [21]. For metastatic model, each BALB/c mouse was given an intravenous injection of 100 μl of cell suspension (4T1-luciferase cells, 4T1-Luc, 1 × 10^5^ cells/ml). Mice were intraperitoneal injected with D-luciferin (Promega, USA) and the in vivo bioluminescence images were obtained dynamically by IVIS Lumina LT (PerkinElmer, USA) at different time point after tumor transplantation. All animals were housed in specific pathogen free conditions with a 12 h/12 h light/dark cycle at 22℃ and unrestricted access to food and water. All relevant mice were randomly divided into two groups (Control, Restraint stress (Stress) or Unpredictable stress (Un-pre), 5 mice in each group).

The restraint-stress procedure was performed based on previously report [22]. In general, tumor-bearing mice were restrained in well-ventilated fixing apparatus for 6 h per day, not allowing forward and backward movement but avoiding compression and hurt. During the restraint-stress procedure, relevant mice in the control group were restricted to food and water, to control variables on hunger and thirsty with mice in stress group.

To establish the unpredictable stress model, mice were exposed to unpredictable physical stressors, such as cage with gradient, wet padding, padding from aggressor, etc. [23]. These discomfort events were last for 6 h per day and were performed without regularity.

### Behavior tests

The open-field test was well-used in evaluating mice autonomic behavior, exploratory behavior and intensity [4]. Mice were individually placed in the corner of an opaque white box (50 cm×50 cm×50 cm) which placed in a separate room with low-light conditions (5 lx) and silence. A virtual zone (20 cm×20 cm) was assumed in the center of the box. Each mouse was allowed to move freely in the box for 6 min. The position and locomotion of each mouse were tracked, recorded and analyzed with a video tracking system with high-resolution digital cameras (ANY-maze, Stoelting Co., Ltd, USA). The frequency and duration of entering the center zone had also been recorded. After each record, mouse was gently removed from the box. Urine and feces were cleaned up. The box was wiped with 75% ethanol and was allowed to evaporate and dry out thoroughly to remove any remaining smell before each new test.

Splash tests were also performed in a separate room with low-light conditions (5 lx) and silence as described previously [4]. Each mouse was received two sprays of 10% (wt/col) sucrose solution and was gently placed into a new cage for free movement. The grooming behaviors were recorded within 5 min.

### Tissue hematoxylin-eosin (HE) staining and immunofluorescence staining

The lung of mouse was obtained and fixed with 4% paraformaldehyde for 24 h, embedded in paraffin and sectioned at 4 μm thickness. The slices were deparaffinized and rehydrated with dimethylbenzene, ethanol and distilled water in order according to the standard protocol and were stained with hematoxylin and eosin respectively [24].

For immunofluorescence staining, after being deparaffinized and rehydrated, slices were performed antigen retrieval using Tris-EDTA (pH = 9) buffer in thermostatted container at 98 °C for 30 min. Sections were permeabilized with 0.2% Triton X-100 in PBS for 20 min, blocked with 3% BSA (MP Biomedicals, USA ) for 60 min at room temperature and were stained with the following primary antibodies overnight at 4℃: Rabbit Anti-Vesicular Acetylcholine Transporter (VAChT) antibody (Abcam, Cambridge, UK), Tyrosine Hydroxylase (TH) Rabbit mAb (Cell Signaling Technology, USA, ), Rabbit anti-CitH3 antibody (Abcam, Cambridge, UK), rabbit anti-EpCAM antibody (Abcam, Cambridge, UK), Rabbit anti-Ly6G antibody (Servicebio, China) and Rabbit anti-Myeloperoxidase (MPO) antibody (Abcam, Cambridge, UK) and Anti-beta Actin antibody (Abcam, Cambridge, UK). After three times wash with PBS, the tissues were then incubated with Alexa-Fluor-conjugated secondary antibodies.

### Specimen acquisition and processing

After different treatments for 2 weeks or indicated times, mice were sacrificed for the follow-up experiments. All specimens were obtained at 9:00 a.m. to prevent the effects of the circadian rhythm. Peripheral blood was collected from the orbit and stored in heparin anticoagulant tubes. Lung tissues were taken out and connective tissue were carefully removed before cutting into small pieces. The tissues were digested in medium with 1 mg/ml collagenase I, II and IV (Sigma, USA) for 2 h at 37 °C in a thermostatic oscillator. After digestion, the cell suspension was filtered with a 40-μm Nylon mesh (BD FALCON, USA) for subsequent use.

### Lung culture

The lung tissues from mice in different groups at 2 weeks were cut into small pieces by sterile scissors and were cultured in a 6-well plate with 2 ml FBS-free RPMI-1640 medium for 24 h at 37℃ in a humidified incubator under 5% CO_2_. Supernatants were collected for further use.

### Flow cytometry

Lung single cell suspensions were stained with Zombie Red Fixable Viability Kit (BioLegend, San Diego, California, USA) to exclude dead cells. Live cells were stained with the following fluorescein conjugated monoclonal antibodies: CD45-Pacific Blue, CD3-PerCP/Cyanine5.5, CD4-FITC, CD8-PE/Cyanine7, TCRγ/δ-PE, CD11b-FITC, Ly-6G-PerCP/Cyanine5.5, Ly-6 C-PE/Cyanine7, CD19-PE and EpCAM-APC. All antibodies mentioned above were purchased from BioLegend (San Diego, California, USA). Anti-Choline Acetyltransferase (ChAT) polyclonal antibody was purchased from Abcam (Cambridge, UK) and Anti-rabbit IgG(H + L) F(ab’)2 Fragment (Alexa Fluor® 594 Conjugate) was purchased from Cell Signaling Technology (USA). The staining procedure of ChAT was performed according to the recommended protocol of intracellular immunofluorescent staining. In brief, after being stained with the surface markers, cells were washed with cell staining buffer (BioLegend, USA) and fixed with Fixation Buffer (BD Biosciences, USA) in 4℃ for 20 min. Fixed cells were resuspended with 100 μl of Perm/Wash Buffer (BD Biosciences, USA) and stained with Anti-Choline Acetyltransferase polyclonal antibody for 1 h in 4℃. Cells were washed and stained with Alexa Fluor® 594 Conjugated Anti-rabbit IgG (H + L). For the flow cytometry analysis, data were acquired with a FACS Fortessa flow cytometer (BD Biosciences, USA) and analyzed with FlowJo Software (Version 10).

### Isolation of neutrophils

The isolation of neutrophils from bone marrow, or from blood and lung tissues of mice in different treatment groups was performed by the Neutrophil Isolation Kit Mouse (Miltenyi Biotec, Germany) according to the instructions of the manufacturer. In brief, cells were washed twice and resuspended with 200 μl buffer containing 0.5% BSA and 2mM EDTA per 5 × 10^7^ cells. 50 μl of Neutrophil Biotin-Antibody Cocktail were added per test and incubated for 10 min at 4℃. Cells were washed and resuspended in buffer, and 100 μl of Anti-Biotin MicroBeads were added. After incubation for 15 min at 4℃, cells were washed and magnetically separated with LS Columns.

Peripheral blood neutrophils from healthy donors were isolated by using EasySep Direct Human Neutrophil Isolation Kit (StemCell Technologies, USA) following the manufacturer’s instructions as previously reported [25].

Purity of neutrophils was evaluated by FACS using antibodies against Ly-6G (Ly-6G monoclonal antibody, PerCP/Cyanine5.5, BioLegend, USA.) and CD11b (CD11b monoclonal antibody, FITC, BioLegend, USA). The purity of neutrophils was > 95%.

### RNA isolation and quantitative real-time PCR

Mouse lung tissues or neutrophils were collected in tubes with lysing matrix and TRIzol reagent (Invitrogen, USA). By using FastPrep system (MP Biomedicals) according to the instructions of the manufacturer, total RNA was extracted. cDNAs were synthesized using a cDNA synthesis kit (TaKaRa, Japan), amplified with TB Green Premix ExTaq (TaKaRa, Japan) and detected by the ABI 7500 Fast Real-Time system (Applied Biosystems, USA). The mRNA level was normalized to the housekeeping gene β-actin expression level and relative expression of each mRNA was calculated using the 2^− ΔΔCt^ method. Primer sequences are listed in Supplementary Table S1.

### Western blot analyses

Lung single cells and neutrophils from mice in different treatment groups were collected and lysed with pre-cooling RIPA lysis buffer (Sangon Biotech, Shanghai, China). The lysates were centrifuged at 12,000 g for 20 min at 4℃. The supernatants were collected and the protein concentrations were determined by BCA assay (Beyotime, China). Protein samples were fractionated on 12% Tris-glycine gels, followed by proteins transfer onto a PVDF membrane (Millipore, Bedford, MA, USA). The membranes were blocked with 5% non-fat dry milk and then probed with diluted primary antibodies against the following mouse proteins, including anti-choline acetyltransferase antibody (Abcam, Cambridge, UK), anti-vesicular acetylcholine transporter (VAChT) antibody (Abcam, Cambridge, UK), anti-PADI4/PAD4 antibody (Abcam, Cambridge, UK). Goat anti-Rabbit IgG-HRP antibody (Abcam, Cambridge, UK) was used as secondary antibody. Antibody binding was visualized with a chemiluminescent substrate on Azure Biosystems (USA).

### ELISA

ACh in serum and lung cultural supernatants was measured by using commercially available ELISA kits (BioLegend, USA) following the manufacturer’s instructions.

### Multi chemokine array

A proteome profiler mouse chemokine array kit (ARY020, R&D, USA) was used to perform multi chemokine array according to the manufacturer’s instruction. Lung cultural supernatants from mice in control and stress groups were incubated with the array membrane which was already pretreated with capture antibodies to specific target proteins, 25 mouse chemokines were visualized simultaneously using chemiluminescent detection reagents.

### Transwell assay

Peripheral blood neutrophils of tumor-bearing mice in control group at 1 week were separated by magnetic separation as described above. Cells were pre-stained with the fluorescent lipid dye-DIO (Beyotime, China). 1 × 10^5^ DIO-labeled neutrophils were resuspended in serum-free culture medium and added to 24-well Transwell inserts (3.0 μm, Corning, USA). Lung culture supernatants from mice in control and stress groups, medium containing 2ng/ml recombinant murine CXCL2 (Peprotech, USA), lung culture supernatant from mice in stress group with 1 μg/ml neutrolizing anti-mouse CXCL2 (R&D Systems, USA) were added to the remaining receiver wells. After incubation for 60 min, the cells in the receiver wells were observed under a fluorescence microscope.

### Confocal microscopy to examine the effects of ACh on NETosis

**N**eutrophils (1 × 10^6^) derived from healthy human peripheral blood and mouse bone marrow were treated, in 24-well tissue culture plate for 4 h, with different concentrations of ACh (12.5, 25, 50 and 100 μM) in the presence or absence of 10 μM nicotinic ACh receptor (nAChR) antagonist mecamylamine or muscarinic ACh receptor (mAChR) antagonist tiotropium. Neutrophils treated with medium alone or lipopolysacchride (LPS, 1 μg/ml) were served as the blank control and positive control groups. The cells were fixed by 4% paraformaldehyde in PBS overnight at 4 °C, then permeabilized with 0.5% TritonX-100 in PBS for 30 min, and washed three times with PBS. Cells were blocked with 5% BSA, 0.1% Tween 20 in PBS for 30 min at room temperature, and then incubated with rabbit anti-MPO antibodies (Abcam, UK) overnight at 4 °C. Next, neutrophils were gently washed three times with PBS and incubated with goat anti-rabbit IgG antibody (Alex Flour 647 nm) for 2 h at room temperature in the dark. Before staining with 100 ng/ml DAPI, neutrophils were incubated with rabbit anti-NE antibodies (Abcam, UK, 1:200 diluted in 0.1% Tween 20 in PBS) overnight at 4 °C, then washed three times and incubated with Alexa Fluor 488 nm goat anti-rabbit IgG fluorescent secondary antibody, finally washed with PBS and ProLong Diamond Antifade Mountant (Thermo, USA) was added to each coverslip, the coverslips were placed on the slides, and then observed with a confocal fluoresce microscope (FV3000 Olympus, Japan). Images were analyzed with the ImageJ software as described previously [25–27].

### Immunohistochemistry to detect ChAT expression in lung tissues

Paraffin embedded human lung tissues from patients with non-tumor bearing pulmonary disease (15 cases) and breast cancer patients with lung metastasis (13 cases) were collected from the Second Affiliated Hospital of Zhejiang University School of Medicine. Clinicopathological characteristics of patients included in this study were shown in Supplementary Table S2 and Supplementary Table S3, respectively. The research complied with all relevant ethical regulations and was approved by the Ethics Committee of the Second Affiliated Hospital of Zhejiang University School of Medicine.

Immunohistochemistry was performed using standard protocol as reported previously [28]. Briefly, the paraffin sections were deparaffinized through alcohol gradients and rehydrated to water. Antigenic retrieval was performed using Tris-EDTA (pH = 9) buffer in thermostatic bath at 98°C for 30 minutes. The sections were blocked in serum at the room temperature for 1 h, and then incubated with a primary Anti-Choline Acetyltransferase antibody (1:2000, Abcam, UK) overnight at 4°C. On the next day, the sections were washed three times with TBS and then incubated with a peroxidase labeled secondary antibody at the room temperature. The color reaction was performed using the 3,3’-diaminobenzidine solution and counterstained with hematoxylin. Slides were scanned using Pannoramic MIDI (3DHISTECH Ltd) and images were captured through Pannoramic Viewer software (3DHISTECH Ltd). The average optical density and the positive percentage were analyzed by ImageJ software.

### Statistical analyses

Statistical analyses were performed with Prism 9 (GraphPad Software). Results are presented as means ± SD. Unpaired, two-tailed Student’s t-tests (two groups), and non-parametric Mann-Whitney U-tests (when Gaussian distribution was not assumed). For multiple comparisons, non-parametric multiple-comparison tests comparing the mean rank of each group (when Gaussian distribution was not assumed), or one- or two-way ANOVAs followed by Dunnett’s multiple comparisons test for one-way ANOVAs and Sidak’s multiple comparisons test for two-way ANOVAs were used. Statistical significance was defined as *p* < 0.05.

## Results

### Chronic stress promotes lung metastasis of breast cancer and activates parasympathetic nervous system in lungs

**Fig. 1:**
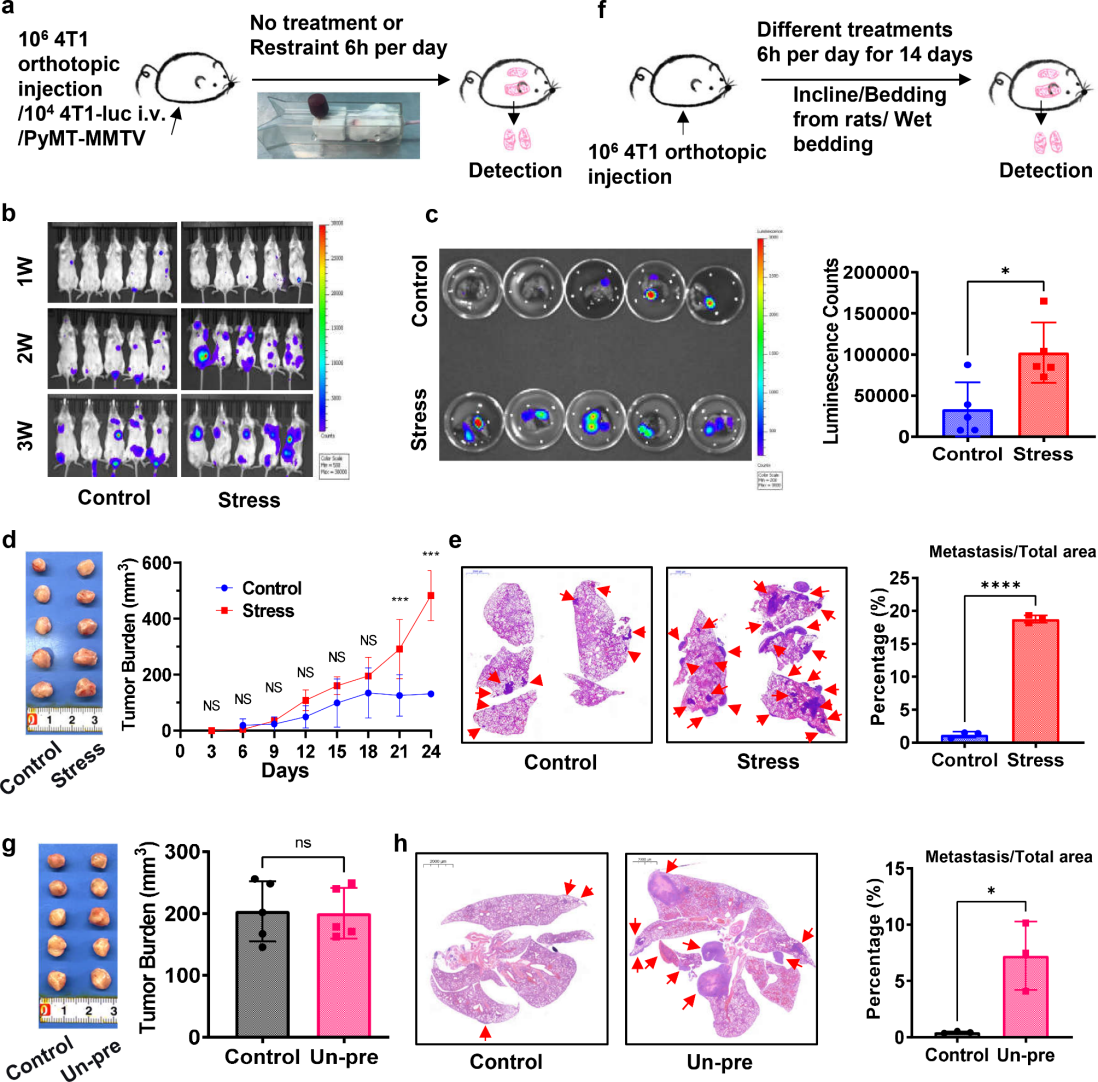
Chronic stress promotes lung metastasis of breast cancer. **a**. Flow chart of the establishment of breast cancer mouse model with chronic restraint stress. **b**. Dynamic observation of tumor burdens by biofluorescence imaging in metastasis model mice. **c**. Fluorescence images of lung tissues in model mice with chronic stress for 3 weeks (left) and the corresponding fluorescence intensity in each group (right). **d**. Photo of primary tumors at 2 weeks (left) and dynamic observation of primary tumor volumes in orthotopic injection mouse model (right). **e**. Histological examination of lungs from orthotopic inoculation model mice at 6 weeks (left) and the percentage of metastatic area to the total lung area (right). Red arrows point to metastatic loci. **f**. Flow chart for the establishment of breast cancer mouse model with chronic unpredictable stress. **g**. Photo of primary tumors (left) and the tumor volumes (right) in orthotopic inoculation mice model with or without unpredictable stress for 2 weeks. **h**. Histological examination of lung tissues from breast cancer mouse model with or without unpredictable stress for 6 weeks (left) and the percentage of metastasis area to total lung area (right). Red arrows point to metastasis of breast cancer in the lung. ns: no sense, *p < 0.05, ***p < 0.001, ****p < 0.0001

To explore the mechanism of chronic stress that promotes lung metastasis of breast cancer, we established three breast cancer-bearing mouse models with two procedures to induce chronic stress. Breast cancer-bearing mice were randomly divided into two groups with or without psychological stress. For restraint stress model (Stress), mice received restraint for 6 h per day (Fig. 1a). For unpredictable stress model (Un-pre), mice received unpredictable stressors (Fig. 1f). The open-field test (Supplementary Fig. S1a-c and e-g) and splash tests (Supplementary Fig. S1d and 1h) were performed to verify the establishment of chronic stress model. In 4T1-Luc metastasis model, breast cancer-bearing mice suffered chronic stress (Stress) metastasized earlier (Fig. 1b) and showed more severe lung metastasis than did mice in control group (Fig. 1b-c). In the orthotopic injection 4T1 breast cancer model, the primary lesion during early stage (< 18 days) was comparable between the two groups (Fig. 1d), however, tumor volumes in the stress group were profoundly larger than those in the control group after 21 days (Fig. 1d). Furthermore, tumor-bearing mice in the stress group showed more severe lung metastases than did mice in the control group (Fig. 1e). The same trend phenomenon could also be observed in the unpredictable stress model. Although there was no significant difference in tumor burdens between the two groups at early stage (Fig. 1g), cancer-bearing mice in the Un-pre group showed more severe lung metastases than did cancer-bearing mice in the control group (Fig. 1h). The phenomenon that chronic stress promotes lung metastasis was verified again in PyMT-MMTV mouse model which was a spontaneous breast cancer mouse model (Supplementary Fig. S2a-c). These data show that chronic stress promotes lung metastasis of breast cancer.

**Fig. 2:**
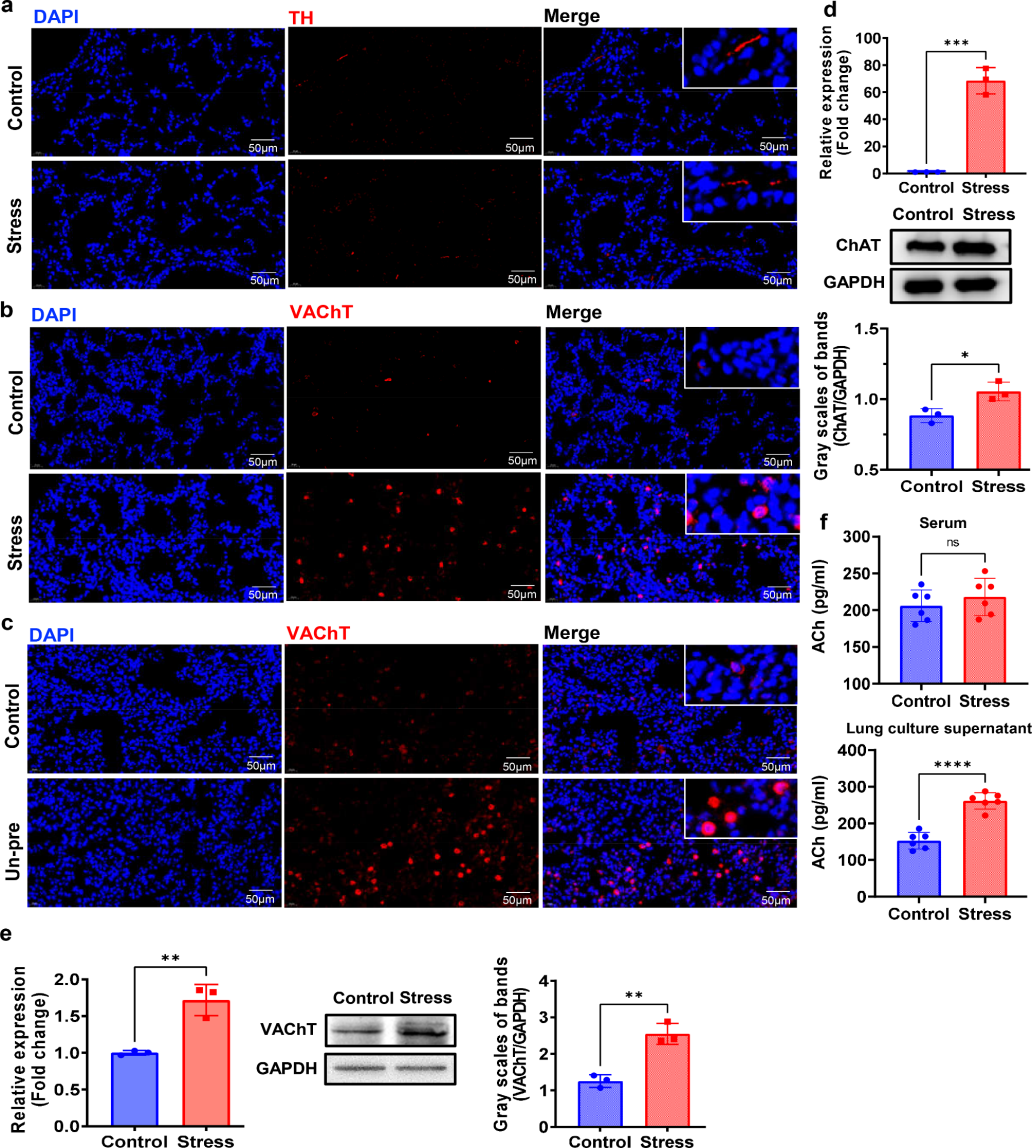
Chronic stress activates parasympathetic nervous system and promotes acetylcholine secretion in lungs. **a-b**. Immunofluorescence examinations of TH (a) and VAChT (b) expression in lung tissues from breast cancer model mice with or without chronic restraint stress for 2 weeks. **c**. Immunofluorescence examination of VAChT expression in lung tissues from breast cancer model mice with or without unpredictable stress for 2 weeks. **d**. qRT-PCR (top) and Western blot analyses of ChAT (middle and bottom) in lung tissues from breast cancer model mice with or without chronic restraint stress for 2 weeks. **e**. qRT-PCR (left) and Western blot analyses (middle and right) of VAChT in lung tissues of mice in control and chronic restraint stress groups at 2 weeks. **f**. ACh concentrations in serum and lung cultural supernatant of breast cancer model mice in control group and chronic restraint stress group for 2 weeks. ns: no sense, *p < 0.05, **p < 0.01, ***p < 0.001, ****p < 0.0001

In reported studies, chronic stress can activate intricate and multiple neuroendocrine changes including the sympathetic nervous system, glucocorticoids and serotonin system [29]. In order to examine the influence of chronic stress on the local activation state of both sympathetic and parasympathetic nerve systems in the lungs of mice with breast cancer, the expression of TH and VAChT, markers of sympathetic and parasympathetic nervous system respectively [30–32], was examined. We found that TH remained unchanged between the control and the stress groups (Fig. 2a), while VAChT was increased significantly in tumor-bearing mice undergone chronic stress (Fig. 2b). The up-regulation of VAChT was also verified in tumor-bearing mice with chronic unpredictable stress (Un-pre) (Fig. 2c). Moreover, the expression of this increased VAChT did not morphologically appear like branches of nerve fibers, but appeared with cell-like shape (Fig. 2b-c), suggesting that the source of this increased VAChT was probably cells other than nerve fibers. We also found that both the transcriptional and protein levels of ChAT and VAChT, which play pivotal roles in the synthesis and transportation of ACh respectively [33], were also elevated in the lungs of tumor-bearing mice with chronic stress (Fig. 2d-e). The content of ACh was significantly increased in culture supernatants of lungs from tumor-bearing mice with chronic stress when compared with that of mice in the control group (Fig. 2f). However, this increase of ACh was restricted to lung tissue because the serum ACh remained comparable between the control and stress groups (Fig. 2f). These data suggest that chronic stress results in activation of parasympathetic nervous system in lung, which might play a role in promoting lung metastasis of breast cancer.

### Chronic stress motivates acetylcholinergic pathway of pulmonary epithelial cells with neuroendocrine functions

VAChTs are members of the solute carrier family 18 (SLC18) of ATP-dependent transporters and can be found in both central and peripheral nervous systems [34, 35]. VAChTs in nerve terminal may show the appearance of branches consistent with the nerves [34]. However, in our study, we found that the increased expression of VAChT in the lung of tumor-bearing mice with stress did not appear like branches of nerve fibers, but morphologically appeared with cell-like shape (Fig. 2b-c), implying that the increased ACh probably derived from cells with neuroendocrine functions other than nerve terminals.

**Fig. 3:**
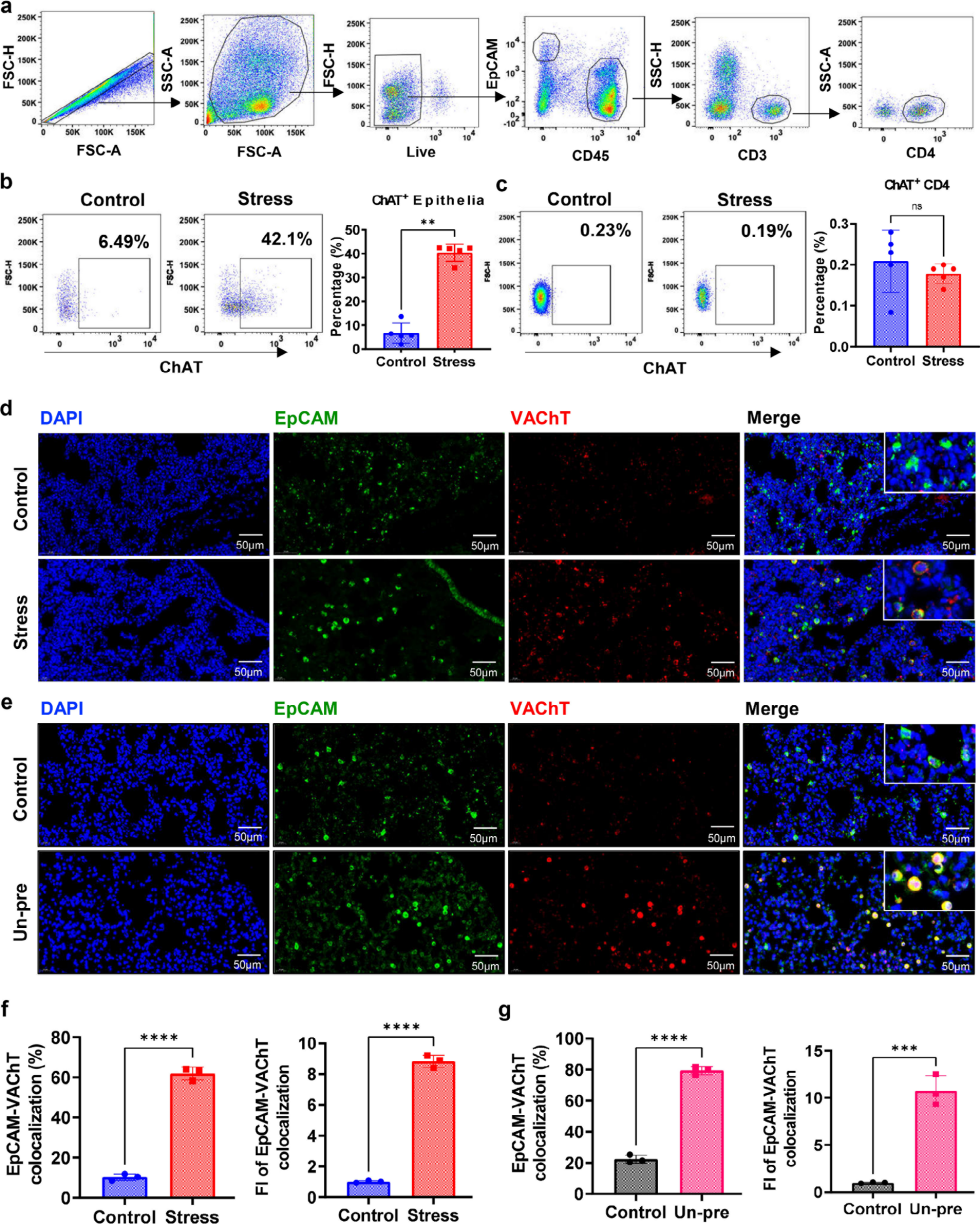
Chronic stress activates acetylcholinergic pathway of pulmonary epithelial cells with neuroendocrine functions. **a**. Gating strategy of flow cytometry analyses of ChAT^+^ epithelial cells and CD4^+^ T cells. **b-c**. FACS analyses of ChAT expression in EpCAM^+^ epithelial cells (b) and CD4^+^ T cells (c) in lungs of breast cancer model mice with or without chronic restraint stress for 2 weeks and their corresponding ChAT positive percentages. **d**. Immunofluorescence examinations of EpCAM and VAChT in lung tissues of breast cancer model mice in control group and chronic restraint stress group at 2 weeks. **e**. Immunofluorescence examinations of EpCAM and VAChT in lung tissues of breast cancer model mice in control group and chronic unpredictable stress group. **f-g.** Percentage (left) and fluorescence intensity (right) of EpCAM-VAChT colocalization in experiments as described in d and e, respectively. ns: no sense, **p < 0.01, ***p < 0.001, ****p < 0.0001

It was reported that lung epithelial cells and splenic CD4^+^ T cells have the ability to synthesize and secrete ACh [33, 36], therefore, the influence of chronic stress on the synthesis of ACh in epithelial cells and CD4^+^ T cells in the lung of tumor-bearing mice was examined. The gating strategy of flow cytometry analyses of ChAT^+^ epithelial cells and CD4^+^ T cells was shown in Fig. 3a. The proportion of ChAT^+^ lung epithelial cells was significantly higher in model mice undergone chronic stress (Fig. 3b), while the expression of ChAT in CD4^+^ T cells remained unchanged (Fig. 3c). This finding was reconfirmed in Un-pre tumor-bearing mice model (Supplementary Fig. S3a-b). Immunofluorescence examination also showed that colocalization of VAChT with EpCAM, a marker of epithelial cells, was profoundly increased in lungs from tumor-bearing mice in both the stress (Fig. 3d, f) and the Un-pre groups (Fig. 3e, g). These data firmly demonstrate that chronic stress motivates acetylcholinergic pathway of pulmonary epithelial cells with neuroendocrine functions and imply that this activation of non-neuroendocrine acetylcholinergic pathway might play a role in the formation of chronic stress promoted pre-metastatic niche of breast cancer in the lung.

### Chronic stress promotes infiltration of neutrophils into lung via CXCL2-CXCR2 axis

**Fig. 4:**
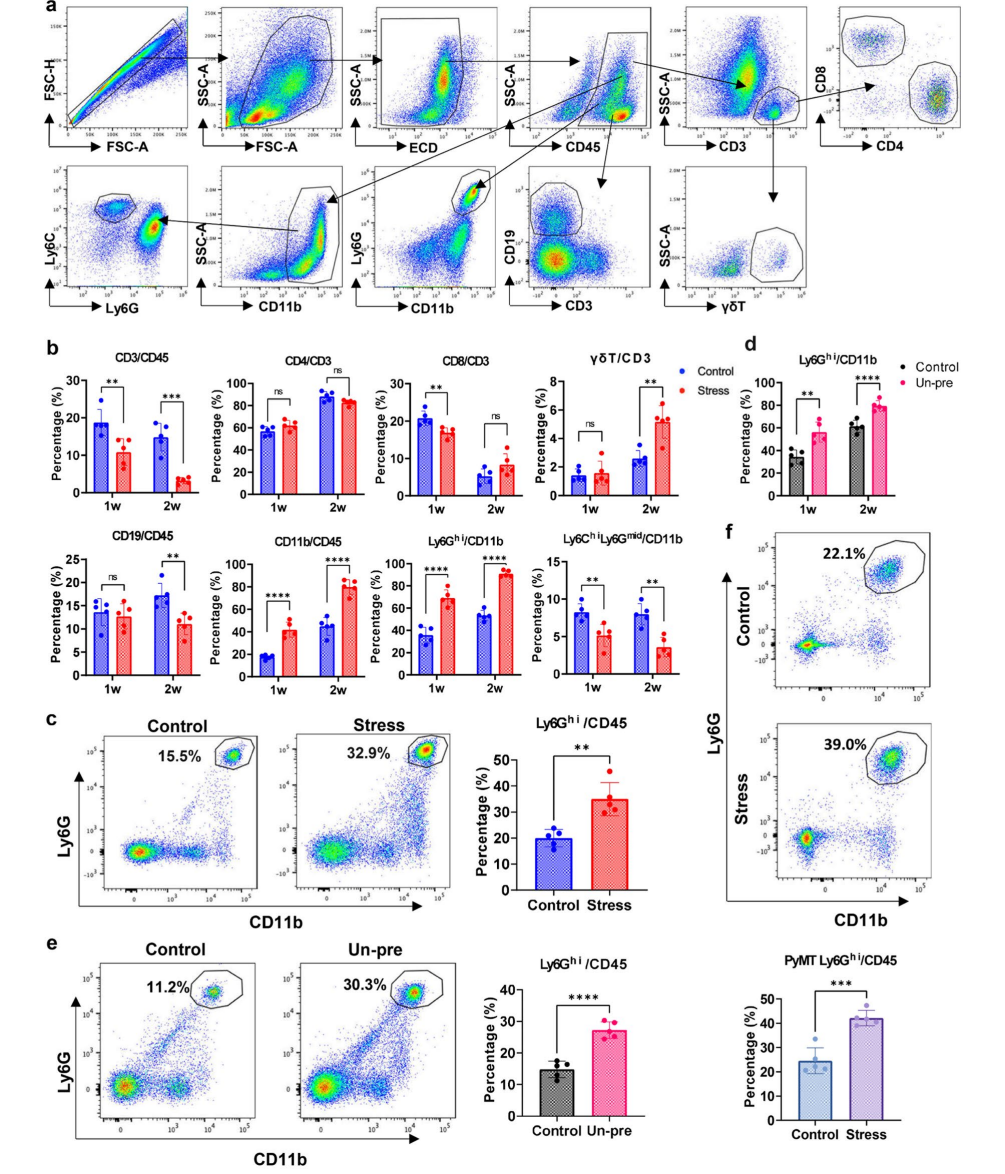
Chronic stress induces infiltration of neutrophils into lung. **a**. Gating strategy of flow cytometry analyses of immune cell subsets. **b**. FACS analyses of immune cell subsets in lungs from orthotopic inoculation murine breast cancer model with or without restraint stress. **c**. FACS analyses of Ly6G^hi^CD11b^+^ neutrophils in lungs from murine breast cancer model with or without chronic restraint stress for 2 weeks. **d**. Percentages of Ly6G^hi^/CD11b^+^ neutrophils in lungs from breast cancer model mice with or without chronic unpredictable stress. **e**. FACS analyses of neutrophils in lungs from model mice with or without chronic unpredictable stress for 2 weeks. **f**. FACS analyses of neutrophils in lungs from 14-week-old PyMT-MMTV spontaneous breast cancer model mice with or without chronic restraint stress for 6 weeks (upper) and their corresponding statistical results (bottom). ns: no sense, **p < 0.01, ***p < 0.001, ****p < 0.0001

The pre-metastatic microenvironment plays a key role in metastasis. The major components of pre-metastatic microenvironment involve immune cells, fibroblasts, epithelial cells, cytokines and other related factors [37, 38]. In order to examine the influence of chronic stress on the pre-metastatic microenvironment in the lung, the immune cell components in lung microenvironment of model mice were analyzed by flow cytometry. The analytic schedule of flow cytometry was shown in Fig. 4a. In orthotopic injection 4T1 breast cancer restraint model, the percentage of T cells (CD3/CD45) decreased significantly along with the time of stress (Fig. 4b). CD4^+^ T cells remained unchanged, while CD8^+^ T cells reduced significantly in stress group at 1 week compared with that of the control group, but remained comparable between the two groups at 2 weeks with a decreased trend along with the progression of the cancer. The percentage of B cells (CD19/CD45) remained unchanged between the two groups at 1 week but decreased in the stress group at 2 weeks. The percentage of γδT cells in the stress group showed a significant increase at 2 weeks. However, myeloid cells (CD11b/CD45) were significantly increased in the lung after stress for 1 week, and neutrophils (Ly6G^hi^) made up the majority of these increased cells (Fig. 4b). Along with the persistent existence of chronic stress (2 weeks), the percentage of neutrophils (Ly6G^hi^/CD11b, or Ly6G^hi^CD11b^+^/CD45) continuously increased (Fig. 4b-c). While another subset of myeloid cells, the macrophages (Ly6C^hi^Ly6G^mid^CD11b^+^), decreased in the stress group compared with that in the control group (Fig. 4b). The phenomenon that chronic stress promotes infiltration of neutrophils into lung of mice with breast cancer was also demonstrated in the Un-pre model (Fig. 4d-e) and in the PyMT-MMTV spontaneous breast cancer mouse model with chronic restraint stress (Fig. 4f).

**Fig. 5:**
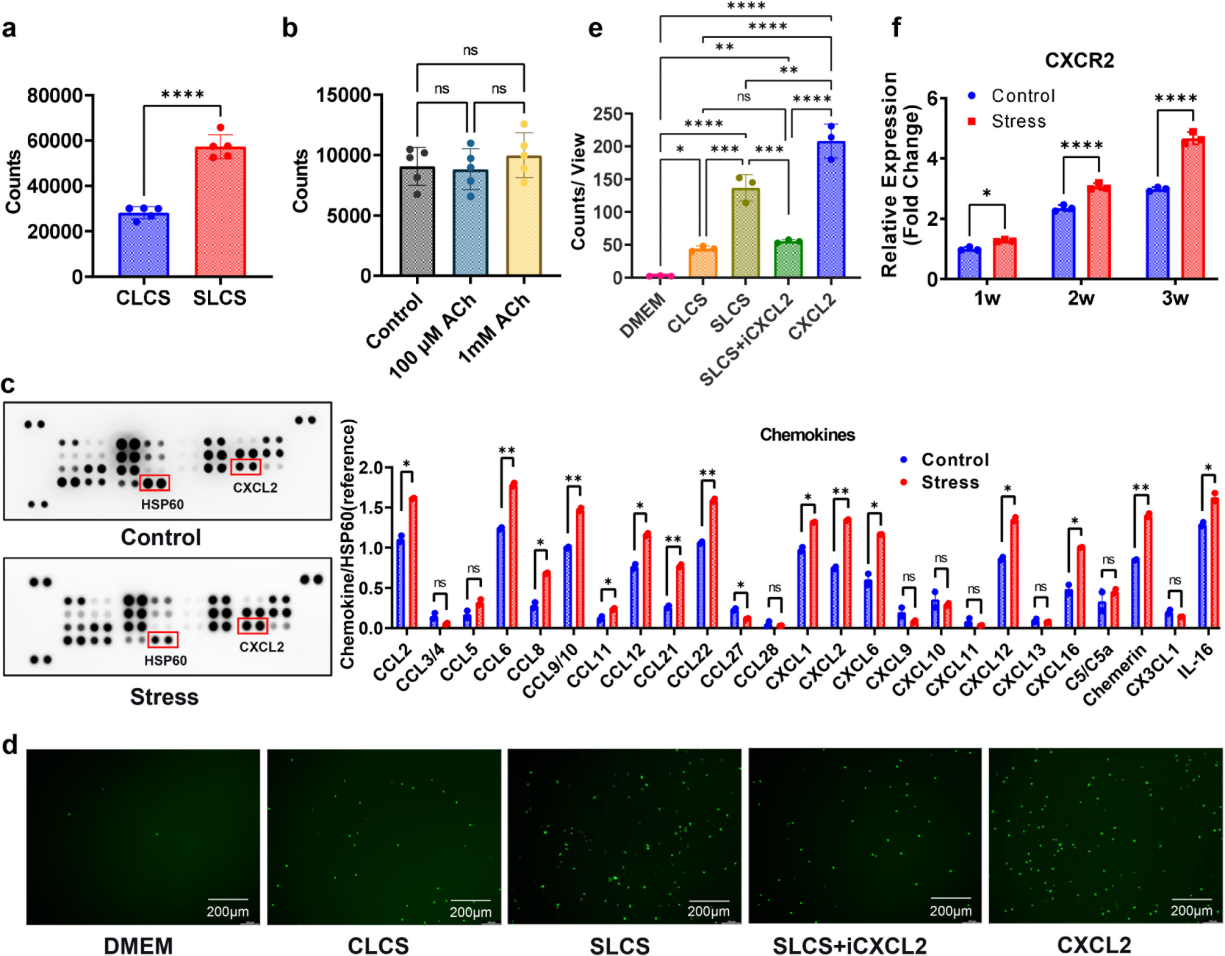
Chronic stress induces the chemotaxis of neutrophils into lung via CXCL2-CXCR2 axis. **a**. Transwell chemotaxis assay to detect the chemotaxis activity of lung culture supernatants to peripheral blood neutrophils of model mice in the control group at 1 week. CLCS: lung culture supernatant of model mice in control group; SLCS: lung culture supernatant of model mice in chronic restraint stress group at 2 weeks. **b**. Transwell chemotaxis assay to detect the chemotaxis activity of ACh to peripheral blood neutrophils from model mice in the control group at 1 week. **c**. Chemokine array to determine the contents of different chemokines in lung culture supernatants from orthotopic inoculation breast cancer mice with or without chronic restraint stress for 2 weeks. **d**. Transwell chemotaxis assay to examine the influence of CXCL2 neutralizing antibody on neutrophil chemotactic activity of SLCS. DMEM: culture medium to serve as the blank control; CLCS: lung culture supernatant of mice in control group; SLCS: lung culture supernatant of mice in chronic restraint stress group at 2 weeks; SLCS + iCXCL2: SLCS + 1 μg/ml CXCL2 neutralizing antibody; CXCL2: 2ng/ml. **e**. Cell counts of experiments as described in d. **f**. qRT-PCR to dynamically detect the mRNA levels of CXCR2 in lung neutrophils from breast cancer model mice with or without chronic restraint stress. ns: no sense, *p < 0.05, **p < 0.01, ***p < 0.001, ****p < 0.0001

Because neutrophils are end differentiated cells, the possibility could be ruled out that the aggregated neutrophils derived from proliferation of neutrophils in the lung microenvironment. To explore the mechanisms by which chronic stress promotes infiltration of neutrophils into the lungs of mice with breast cancer, chemotactic activity of lung culture supernatants from different groups of mice with orthotopic injection of 4T1 breast cancer was examined. Neutrophil chemotactic activity of lung culture supernatants from mice in the stress group (SLCS) was profoundly higher than that of the control group (SCLC) (Fig. 5a). Because chronic stress resulted in elevated ACh in lung culture supernatant (Fig. 2f), the chemoattractant activity of ACh was also examined. However, ACh itself did not show any chemoattractant activity to neutrophils compared to the medium (Control) (Fig. 5b). These data suggest that chronic stress promoted infiltration of neutrophils is probably mediated by chemokines. Therefore, a chemokine array was performed to determine the content of different chemokines in lung culture supernatants. CCL2, CCL6, CCL8, CCL9/10, CCL11, CCL12, CCL21, CCL22, CXCL1, CXCL2, CXCL6, CXCL12, CXCL16, Chemerin and IL-16 had a higher expression in lung culture supernatants from mice in the stress group compared with those in control group (Fig. 4c). Because CXCL2 is one of the most powerful chemokines for neutrophils [24], and it was also one of the most significantly increased chemokines in the lung culture supernatants of cancer-bearing mice with chronic stress, we next observed the influence of CXCL2 neutrolization on the neutrophil chemotactic activity of lung culture supernatants. Our data showed that the stress enhanced neutrophil chemoattractant activity of lung culture supernatant could be significantly attenuated by CXCL2 neutrolizing antibody (Fig. 5d-e). We also observed the impact of chronic stress on the expression of CXCR2, one of the receptors for CXCL2, and found that chronic stress promoted CXCR2 expression in neutrophils in the lung (Fig. 5f). These data suggest that chronic stress promotes infiltration of neutrophils into lung of mice with breast cancer mainly via CXCL2-CXCR2 axis.

### Chronic stress enhances formation of neutrophil extracellular traps in the lung and promotes NETotic neutrophils to capture breast cancer cells

**Fig. 6:**
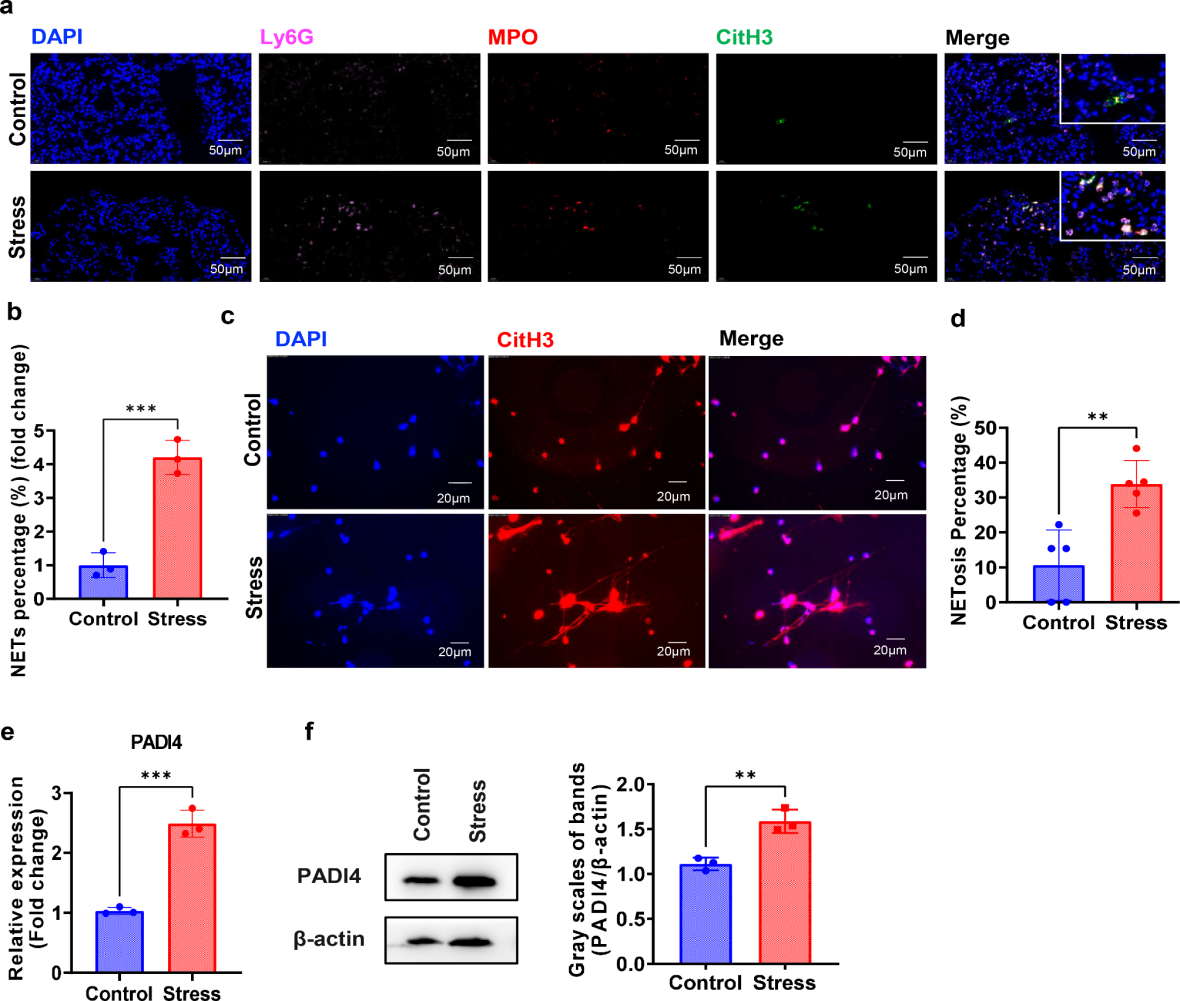
Chronic stress enhances NETosis of neutrophils in the lung of model mice. **a**. Immunofluorescence analyses of in situ NETosis of neutrophils in lung tissues of breast cancer model mice with or without chronic restraint stress for 2 weeks. **b**. NETosis percentages of neutrophils in experiments as described in a. **c**. Immunofluorescence to examine NETs structures of neutrophils isolated from lungs of model mice in control group and in chronic restraint stress group at 2 weeks. **d**. NETosis percentages of neutrophils in experiments as described in c. **e-f**. qRT-PCR and Western blot to detect mRNA (e) and protein (f) levels of PADI4 in neutrophils isolated from lungs of model mice in control group and in chronic restraint stress group at 2 weeks. **p < 0.01, ***p < 0.001

Neutrophil extracellular traps (NETs) formation or NETosis has been reported to participate in the occurrence and metastasis of cancer [39, 40]. The influence of chronic stress on NETosis of the recruited neutrophils in the lung was examined. The in situ NETosis was significantly enhanced in the stress group of mice with breast cancer compared with that in the control group (Fig. 6a-b). This finding was further confirmed by in vitro experiments. Neutrophils isolated from lung tissues of tumor-bearing mice in the stress group showed higher ability to undergo NETosis than did neutrophils from the control group (Fig. 6c-d). Peptidylarginine deiminase 4 (PADI4) is a key enzyme that induces citrullination of histone arginine residues, hereby leading to the depolymerization of chromatin in NETosis [25, 39], which also serves as an indicator of NETosis. Our data showed that neutrophils sorted from lungs of tumor-bearing mice in the stress group significantly up-regulated the expression of PADI4 at both transcriptional and protein levels (Fig. 6e-f). These data indicate that chronic stress enhances NETosis of neutrophils in the lung of mice with breast cancer.

**Fig. 7:**
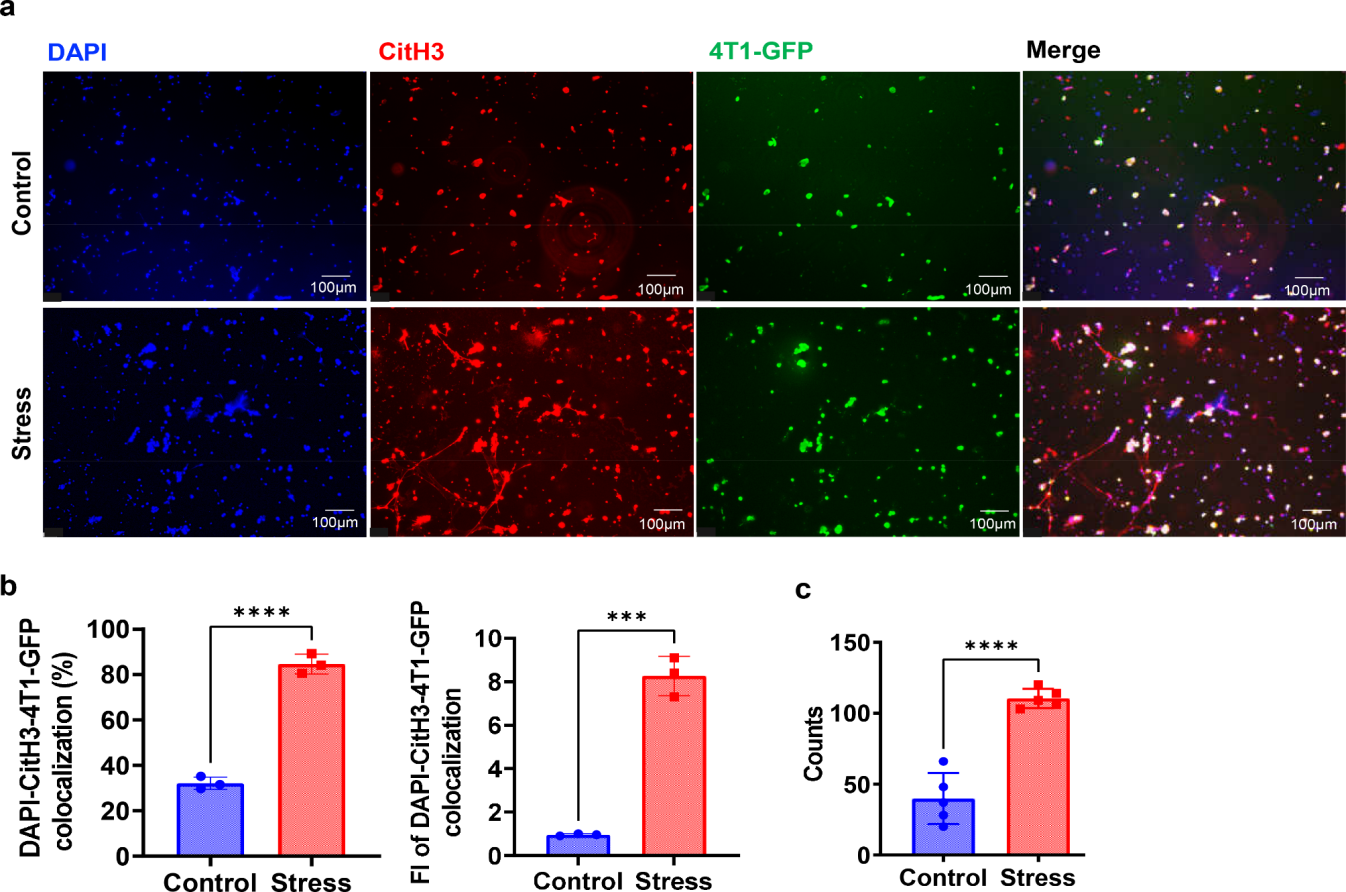
Chronic stress promotes NETotic neutrophils to capture breast cancer cells. **a**. Confocal analyses to examine the ability to capture cancer cells of neutrophils isolated from lungs of model mice with or without chronic restraint stress for 2 weeks. **b**. Percentage (left) and fluorescence intensity (fold change, right) of DAP-CitH3-4T1-GFP colocalization in experiments as described in a. **c**. Number of 4T1-GFP cells captured by NETotic neutrophils in experiments as described in a. ***p < 0.001, ****p < 0.0001

NETs can not only capture circulating tumor cells (CTCs) to prolong the stay duration of CTCs, but also facilitate the migration and invasion abilities of CTCs, hereby promoting tumor metastasis [39, 40]. To observe the influence of stress on the ability of NETotic neutrophils to capture the cancer cells, confocal analyses were carried out. Neutrophils derived from lungs of mice in the stress group captured more 4T1-GFP cells than did neutrophils from the control group (Fig. 7a-c), implying that chronic stress promotes NETotic neutrophils to arrest cancer cells in the lung.

### Acetylcholine enhances NETosis of neutrophils from human and mouse

**Fig. 8:**
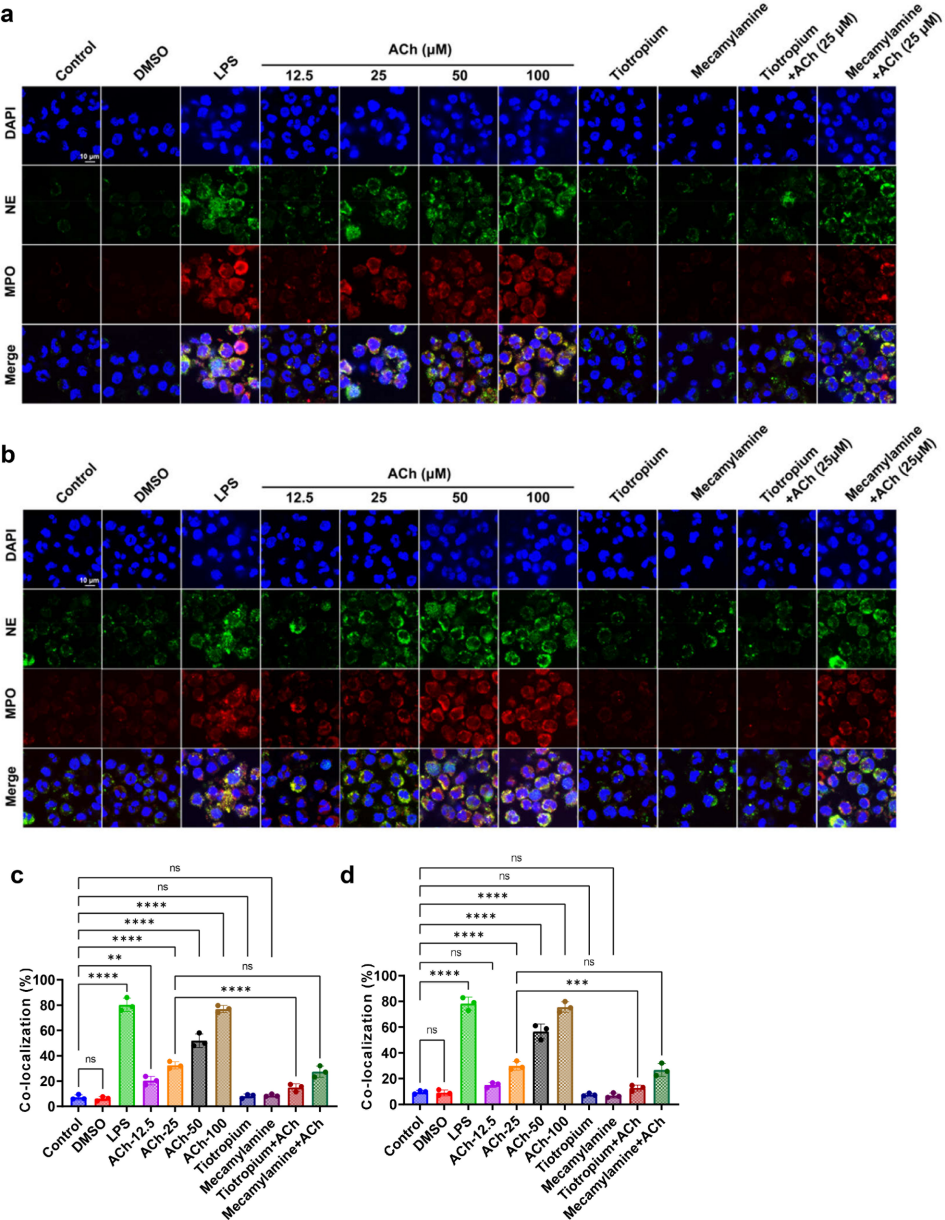
ACh promotes NETosis of neutrophils from healthy human and mice. **a-b**. Confocal analyses to examine the effects of ACh on NETosis of healthy human peripheral blood neutrophils (a) and mouse bone marrow derived neutrophils (b). **c-d**. NETosis percentage of human (c) and mouse (d) neutrophils in different treatment groups, respectively. ns: no sense, **p < 0.01, ***p < 0.001, ****p < 0.0001

Because chronic stress promotes NETosis (Fig. 7) and induces lung epithelial cells to produce ACh (Fig. 2f), the influence of ACh on NETosis was examined. ACh-treated neutrophils derived from lung of tumor-bearing mice in the control group showed significantly increased NETosis compared to that without ACh treatment (Supplementary Fig. S4). Furthermore, we also examined the influence of ACh on the NETosis of neutrophils from healthy human and mouse. ACh dose-dependently enhanced NETosis of neutrophils from both healthy human (Fig. 8a, c) and mouse (Fig. 8b, d). In order to further examine what type of ACh receptor mediates the enhancement effect of ACh on NETosis, the nicotinic ACh receptor (nAChR) blocker mecamylamine and muscarinic ACh receptor (mAChR) antagonist tiotropium were applied. Tiotropium but not mecamylamine could abrogate the ACh enhanced NETosis (Fig. 8), suggesting that mAChR is the receptor mediating the NETosis promoting effects of ACh.

### Breast cancer patients with lung metastasis express increased ChAT in their lungs

**Fig. 9:**
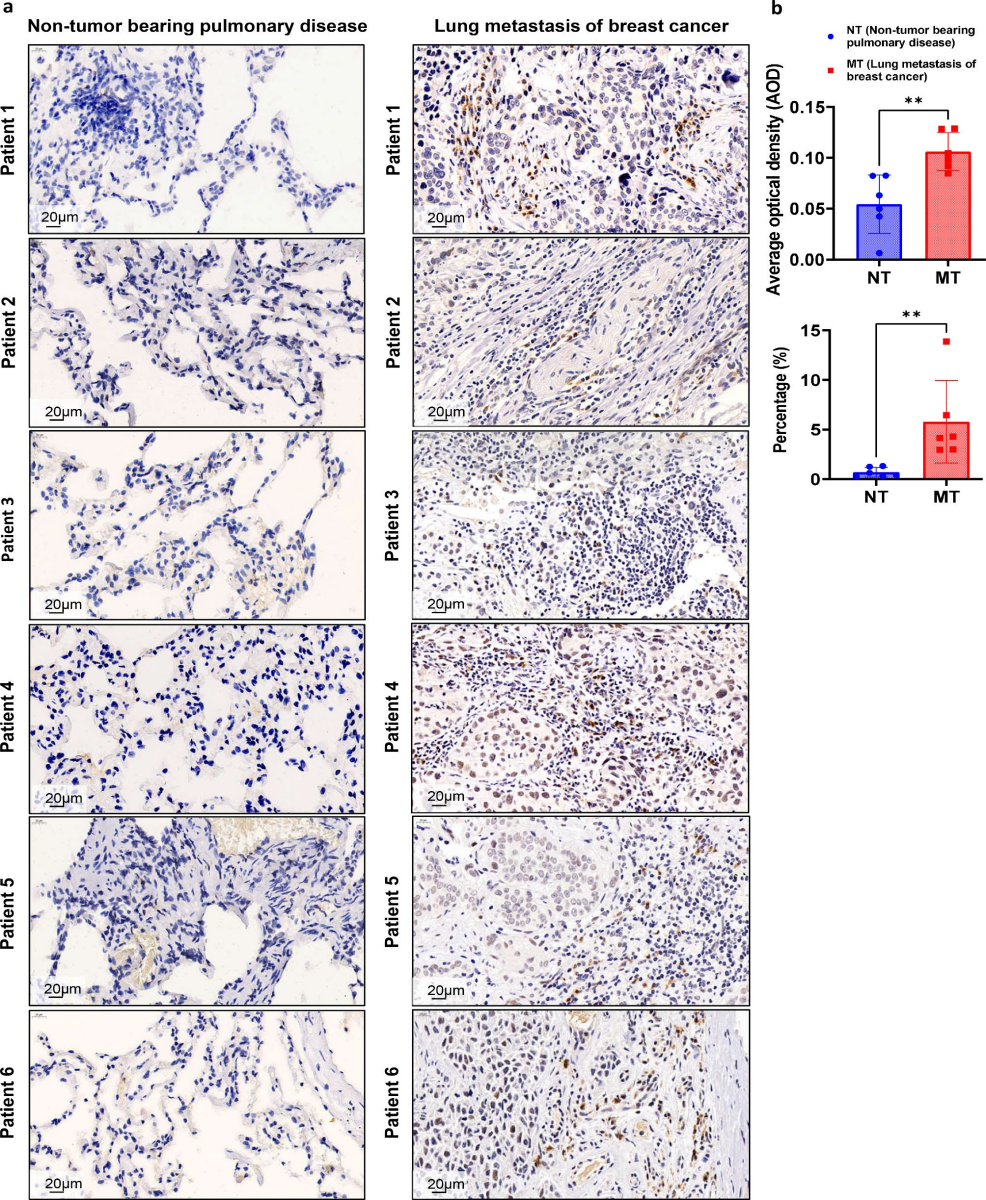
Immunohistochemistry analyses of ChAT expression in lung tissues of breast cancer patients with lung metastasis. **a**. Representative immunohistochemistry images of ChAT expression in lung tissues from patients with non-tumor bearing pulmonary diseases and from breast cancer patients with lung metastasis. **b**. Average optical density (upper) and percentage of ChAT positive area to total area (bottom) analyzed by ImageJ software. **p < 0.01

In the biosynthesis and secretion of ACh, extracellular choline is imported via high affinity choline transporter CHT1; in the presence of mitochondria-derived acetyl-CoA, choline is catalyzed into ACh by ChAT in the cytoplasm. The ACh is then packaged into vesicles and released via VAChT in the manner of exocytosis [33]. Therefore, the expression of ChAT and VAChT correlates well with the ability to produce ACh. The expression of ChAT in lung tissues from breast cancer patients with lung metastasis and from patients with non-tumor bearing pulmonary diseases was examined by immunohistochemistry. Six representative results in each group were shown in Fig. 9. Breast cancer patients with lung metastasis showed significantly increased expression of ChAT in their lungs than did patients with non-tumor pulmonary diseases. These data suggest the important role of ACh in remodeling of lung pre-metastatic niche of patients with breast cancer.

## Discussion

In recent years, significant progress has been achieved in the study of the mechanisms underlying chronic stress promoted metastasis of breast cancer. Most studies focused on the role of activation of sympathetic nervous system and the changes of other stress-related hormones in the peripheral circulation in metastasis [15, 16, 41–43]. These stress factors promote most hallmarks of cancer through impacting the malignant tissues, their microenvironment, immunity, lymphatic flow and distant potential pre-metastatic niches [44]. High levels of glucocorticoids may induce carcinogenesis through inhibition of the nuclear erythroid factor 2-related factor 2 (Nrf2) mediated protective mechanism [45, 46]. However, the function and organ specificity of chronic stress in shaping the pre-metastatic niche remain largely unknown.

In this study, we found that non-neurogenic ACh participated in remodeling of the pre-metastatic microenvironment in chronic stress induced lung metastasis of breast cancer.

Early in 2013, Paul S Frenette and his colleagues have demonstrated that both the sympathetic nervous system and parasympathetic nervous system are innervated in tumor microenvironment of prostate cancer [32]. The sympathetic nervous system was involved in tumorigenesis, whereas the parasympathetic nervous system participated in the metastasis of tumor [32]. However, there are few studies talking about whether chronic stress would regulate the parasympathetic nervous system. In a mouse model of prostate cancer, the use of nonselective mAChR agonist carbachol could facilitate the invasion and metastasis of mouse prostate tumor, while nonselective mAChR antagonist scopolamine or mAChR1 gene specifically knockout could rescue this unpleasant event [32]. Besides mAChR, another subtype α9 of nAChR, which interacts with ERBB2 and epidermal growth factor receptor, can also influence intercellular adhesion of breast tumor cells thus mediating the metastasis of breast tumor [47]. These findings suggest that parasympathetic nervous system also plays a non-negligible role in tumor metastasis.

We demonstrated, for the first time, that chronic stress could activate parasympathetic nerve system in the lung of breast cancer model mice, leading to the elevation of ACh in the lung (Fig. 2). The increased expression of VAChT, one of the parasympathetic nerve system markers, did not morphologically appear like branches of nerve fibers, but appeared with cell-like shape (Fig. 2b-c), suggesting that the source of this increased ACh was probably cells other than nerve fibers. It was reported that pulmonary neuroendocrine cells and immune cells such as CD4^+^ T cells had the ability to secrete non-neuronal ACh, which played important roles in pathophysiological activities [48–53]. By using immunofluorescence and flow cytometry, we demonstrated the expression of ChAT was up-regulated in pulmonary epithelial cells rather than CD4^+^ T cells (Fig. 3), confirming that the source of this elevated ACh is pulmonary epithelial cells.

Besides the direct regulatory effects on the metastasis-related properties of tumor cells, chronic stress can also mediate the dysfunction of immune system and promote the metastasis of tumor [44, 54]. The dysfunction of immune system includes the abnormal development and differentiation of immune cells, overactivation of immunoreaction, metabolic disorders, imbalance between immunosuppressive cells and anti-tumor cells [54]. It has been reported that chronic stress promotes metastasis via the polarization of macrophages to type II macrophages (M2), which can produce more prostaglandin E2, up-regulate the expression of VEGF and induce the neovascularization in situ [15, 42]. Meanwhile, chronic stress can also promote macrophages towards distant organs, such as lung and lymph nodes, to remodel the pre-metastatic niche [41]. Other researchers have also found that NK cells are involved in chronic stress-induced immunoregulation and the attenuated killing ability of NK cells leads to the metastasis of tumor [55]. To further confirm the influence of chronic stress on lung pre-metastatic niche, immune cell subsets were analyzed. We found that chronic stress could remodel lung pre-metastatic niche with striking increase in neutrophil infiltrations in breast cancer model mice (Fig. 4). Furthermore, we demonstrated that chronic stress could enhance production of chemokines such as CXCL2, and the neutrophil chemotaxis activity of lung culture supernatants from model mice in stress group could be attenuated significantly by CXCL2 neutrolizing antibody (Fig. 5). These data demonstrate that these infiltrated neutrophils are mainly recruited by stress induced CXCL2.

It is well known that NETosis is closely related to metastasis of tumor. CTCs can be captured by NETs, and the prolonged adhesion and stay in distant organs offer a much greater chance to form a successful metastasis [39, 40]. In the meantime, NETs can also promote the migration and invasion of tumor cells, reactivate dormant tumor cells in distant organs and form tumor metastasis [56]. NET trapping of cancer cells was associated with increased formation of hepatic micrometastases and gross metastatic disease burden in a murine lung cancer model [57]. By using intravital imaging, Park et al. demonstrated that NET-like structures around metastatic 4T1 cancer cells that had reached the lungs of mice, and treatment with NET-digesting, DNase I-coated nanoparticles markedly reduced lung metastases in mice [58]. Moreover, it was also reported that NET-DNA could act as a chemotactic factor to attract cancer cells, rather than merely acting as a “trap” for them, in several mouse models [59]. In our studies, we found that chronic stress enhanced both in situ and in vitro NETosis of neutrophils in the lung of breast cancer model mice (Fig. 6), and promoted these NETotic neutrophils to capture cancer cells (Fig. 7). We also showed that ACh could promote NETosis of neutrophils isolated from lungs of breast cancer model mice in the control group (Supplementary Fig. S4). This NETosis enhancing effect of ACh was confirmed again when neutrophils from healthy human and mouse were applied (Fig. 8). These data demonstrate that NETosis in pre-metastatic niche which is promoted, at least in part, by chronic stress induced ACh plays an important role in lung metastasis of breast cancer.

Because ChAT and VAChT are key enzymes involved in the biosynthesis of ACh, the expression of ChAT and VAChT correlates well to the ability of cells to produce ACh [33]. Immunohistochemistry analyses showed that expression of ChAT in lung tissues of breast cancer patients with lung metastasis was significantly higher than that expressed in patients with non-tumor pulmonary diseases (Fig. 9), suggesting the role of ACh in remodeling of lung pre-metastatic niche in clinical settings.

**Fig. 10:**
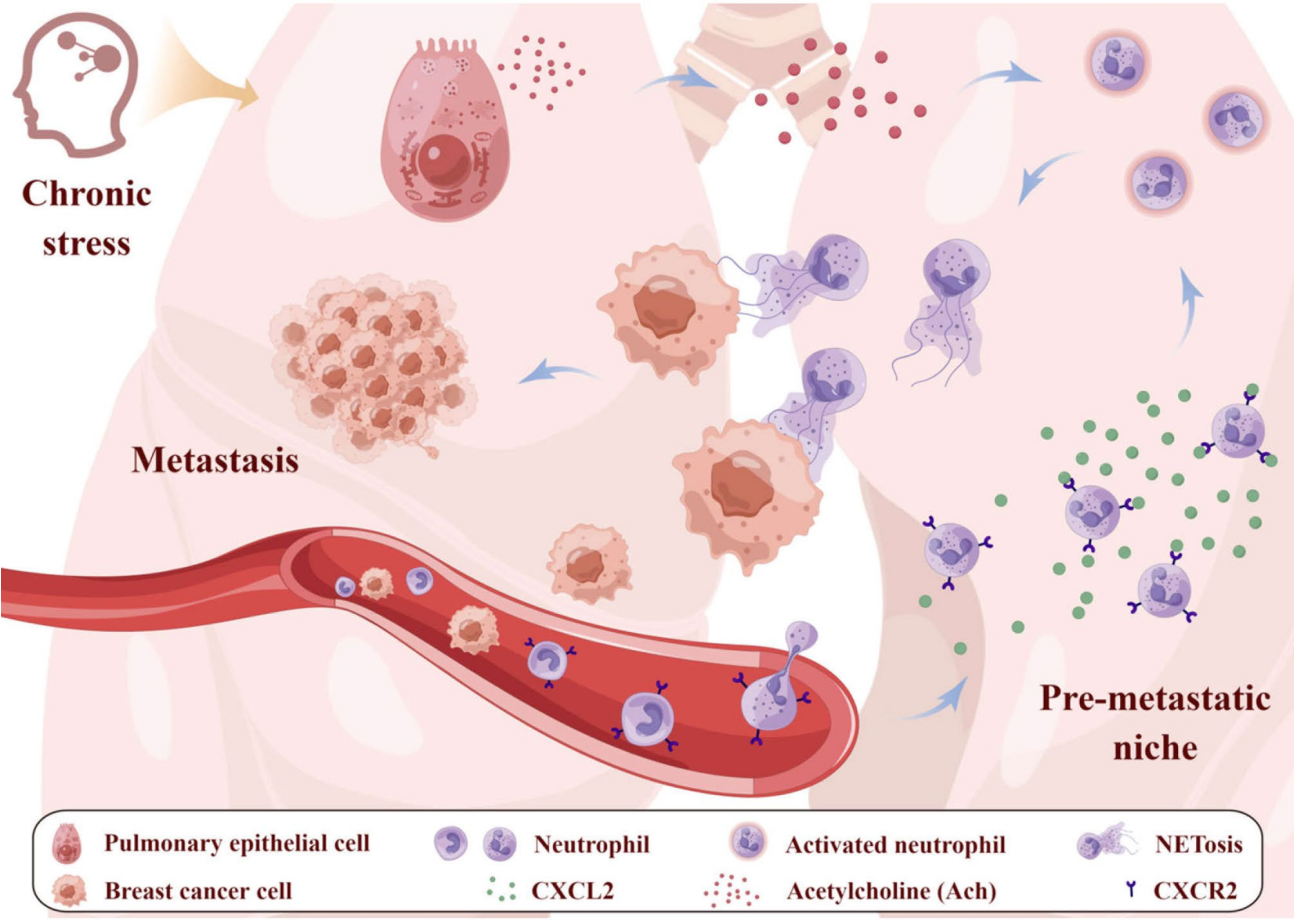
Schematic diagram of the mechanisms by which chronic stress remodels lung pre-metastatic niche of breast cancer. Chronic stress remodels lung pre-metastatic niche of breast cancer mainly by two ways. On one hand, chronic stress promotes production of CXCL2 chemokine in the lung, which recruits neutrophils. On the other hand, chronic stress motivates lung epithelial cells to secrete ACh that enhances NETosis of the aggregated neutrophils in the lung. The NETotic neutrophils capture the circulating tumor cells, hereby favoring lung metastasis of breast cancer. This image (AARIT33ae3) was generated by Figdraw (https://www.figdraw.com)

In summary, this study demonstrates that chronic stress remodels lung pre-metastatic niche of breast cancer by recruiting neutrophils into lung and activating pulmonary epithelial cells to secrete ACh that promotes NETosis (Fig. 10). Our findings provide not only a novel mechanism by which chronic stress promotes lung metastasis of breast cancer, but also new clues in the design of treatment modality of breast cancer. However, the following questions need to be answered in the future. What is the molecular mechanism by which chronic stress activates acetylcholinergic pathways in pulmonary epithelial cells? Which subset of lung epithelial cells can be activated to produce ACh under chronic stress and its related mechanisms? What is the cell source of CXCL2 under chronic stress and its related mechanisms? What are the molecular mechanisms underlying ACh promoted NETosis of neutrophils? Along with the illumination of these questions, the mechanisms by which chronic stress promotes lung metastasis of breast cancer will be further clarified, which helps definitely the development of targeted precision prevention of lung metastasis in patients with breast cancer.

## Conclusions

This study investigated the role of chronic stress in the remodeling of lung pre-metastatic niche of breast cancer. We demonstrated, for the first time, that chronic stress motivates cholinergic pathway in pulmonary epithelial cells, leading to significant increase of ACh in lung culture supernatants of model mice with chronic stress. Chronic stress remodels the pre-metastatic niche with striking increase of neutrophils in the lung. Chronic stress induced CXCL2 is the key chemokine that recruits neutrophils into the lung. Meantime, chronic stress enhances in situ NETosis of neutrophils in the lung and promotes NETotic neutrophils to capture the cancer cells. In vitro experiments showed that NETosis of neutrophils from lung of model mice in the control group and from healthy human and mouse can also be enhanced by ACh. More importantly, the expression of ChAT in lung tissues from breast cancer patients with lung metastasis is profoundly higher than that in lungs of patients with non-tumor pulmonary diseases. Taken together, we demonstrated that chronic stress remodels lung pre-metastatic niche of breast cancer by enhancing expression of CXCL2 that recruits neutrophils into lung and by activating epithelial cells to produce ACh that promotes NETosis of neutrophils.

### Electronic supplementary material

Below is the link to the electronic supplementary material.

**Additional file 1: Supplementary Fig. S1**. Behavior tests to identify breast cancer mouse model with chronic stress. **a**. Open field test results of orthotopic inoculation breast cancer mouse model with or without chronic restraint stress. Representative path diagrams of 3 mice in each group were shown. **b-c**. Total distance (b) and average speed (c) of a single mouse within 6 min in experiments as described in a. **d**. Sugar water splash test results. Total time of model mice licking sugar water in control and chronic restraint stress groups within 5 min was shown. **e**. Open field test results of mice in control group and in chronic unpredictable stress group. Representative path diagrams of 3 mice in each group were shown. **f-g**. Total distance (f) and average speed (g) of a single mouse within 6 min in experiments described in e. **h**. Total time of model mouse licking sugar water in the control and chronic unpredictable stress groups within 5 min was shown. **p < 0.01, ***p < 0.001

**Additional file 2: Supplementary Fig. S2**. Chronic stress promotes long metastasis of PyMT- MMTV spontaneous breast cancer mouse model. **a**. Primary tumor weights in 14-week-old PyMT-MMTV spontaneous breast cancer model mouse with or without chronic stress for 6 weeks. **b**. Histological examination of lung tissues from PyMT-MMTV spontaneous breast cancer model mouse with or without chronic stress for 6 weeks. Red arrows point to the metastatic loci. **c**. Percentages of metastasis area to the lung total area. ns: no sense, **p < 0.01

**Additional file 3: Supplementary Fig. S3**. ChAT expression in pulmonary epithelial cells in model mice with chronic unpredictable stress. **a**. FACS analysis of ChAT expression in EpCAM^+^ epithelial cells in lungs of model mice with or without chronic unpredictable stress for 2 weeks. **b**. Mean fluorescence intensity of ChAT in pulmonary epithelial cells of model mice in the Control and Un-pre groups. **p < 0.01

**Additional file 4: Supplementary Fig. S4**. ACh promotes NETosis of neutrophils isolated from lungs of 4T1 orthotopic injection breast cancer model mice in the control group. **a**. 1 × 10^4^ neutrophils isolated from lungs of model mice in the control group at 1 week were treated with 100 μM ACh for 4 h. NETosis was examined by immunofluorescence. **b-c**. Percentage of NETosis (b) and fold change of fluorescence intensity of colocalization (c). **p < 0.01; ***p < 0.001

**Additional file 5: Supplementary Table S1**. Primers used in qRT-PCR

**Additional file 6: Supplementary Table S2**. Clinicopathological characteristics of female breast cancer patients with lung metastasis

**Additional file 7: Supplementary Table S3**. Clinical information of non-tumor patients with pulmonary diseases

## Data Availability

Data supporting the findings of this manuscript are available from the corresponding author upon request. Full scan images of the Gels, Blots and Source data for figures and numbers are provided with this paper.

## Abbreviations

ACh: Acetylcholine
ChAT: Choline acetyltransferase
CLCS: Lung culture supernatant from control group
CTC: Circulating tumor cells
EMT: Epithelial-mesenchymal transition
LPS: Lipopolysaccharide
MPO: Myeloperoxidase
NE: Neutrophil elastase
NETs: Neutrophil extracellular traps
OS: Overall survival
PFS: Progression-free survival
SLCS: Lung culture supernatant from stress group
TH: Tyrosine hydroxylase
VAChT: Vesical acetylcholine transferase
VEGF: Vascular endothelial growth factor
