# Supplementary figures and images for: Chronic stress induces pulmonary epithelial cells to produce acetylcholine that remodels lung pre-metastatic niche of breast cancer by enhancing NETosis
Source: J Exp Clin Cancer Res. 2023 Sep 29;42:255. doi: 10.1186/s13046-023-02836-5 (PMC10540414; doi:10.1186/s13046-023-02836-5)

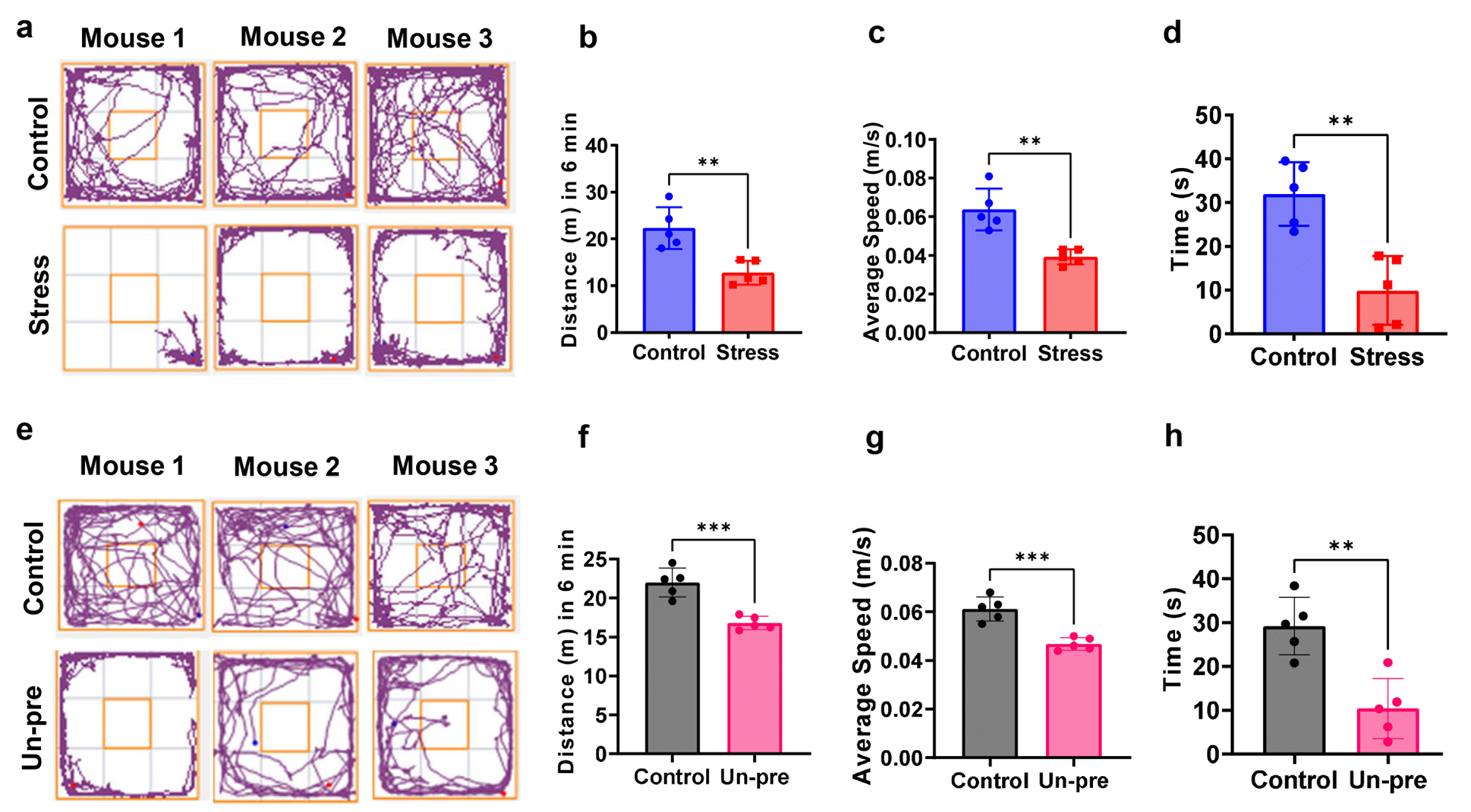

Supplement: Supplementary file 1 — Additional file 1: Supplementary Fig. S1. Behavior tests to identify breast cancer mouse model with chronic stress. a. Open field test results of orthotopic inoculation breast cancer mouse model with or without chronic restraint stress. Representative path diagrams of 3 mice in each group were shown. b-c. Total distance (b) and average speed (c) of a single mouse within 6 min in experiments as described in a. d. Sugar water splash test results. Total time of model mice licking sugar water in control and chronic restraint stress groups within 5 min was shown. e. Open field test results of mice in control group and in chronic unpredictable stress group. Representative path diagrams of 3 mice in each group were shown. f-g. Total distance (f) and average speed (g) of a single mouse within 6 min in experiments described in e. h. Total time of model mouse licking sugar water in the control and chronic unpredictable stress groups within 5 min was shown. **p < 0.01, ***p < 0.001 [file 13046_2023_2836_MOESM1_ESM.png]

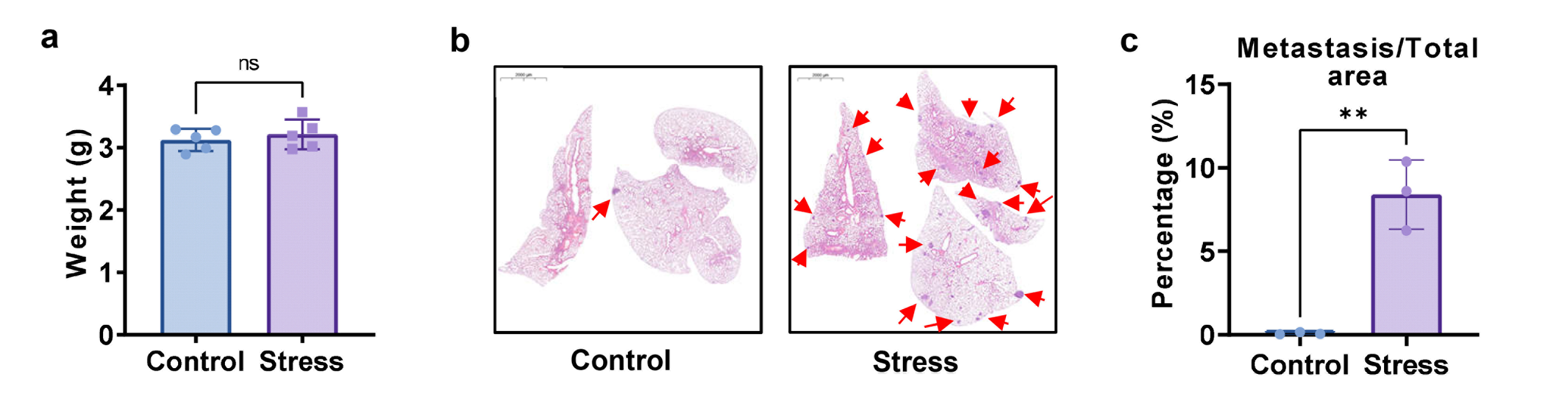

Supplement: Supplementary file 2 — Additional file 2: Supplementary Fig. S2. Chronic stress promotes long metastasis of PyMT- MMTV spontaneous breast cancer mouse model. a. Primary tumor weights in 14-week-old PyMT-MMTV spontaneous breast cancer model mouse with or without chronic stress for 6 weeks. b. Histological examination of lung tissues from PyMT-MMTV spontaneous breast cancer model mouse with or without chronic stress for 6 weeks. Red arrows point to the metastatic loci. c. Percentages of metastasis area to the lung total area. ns: no sense, **p < 0.01 [file 13046_2023_2836_MOESM2_ESM.png]

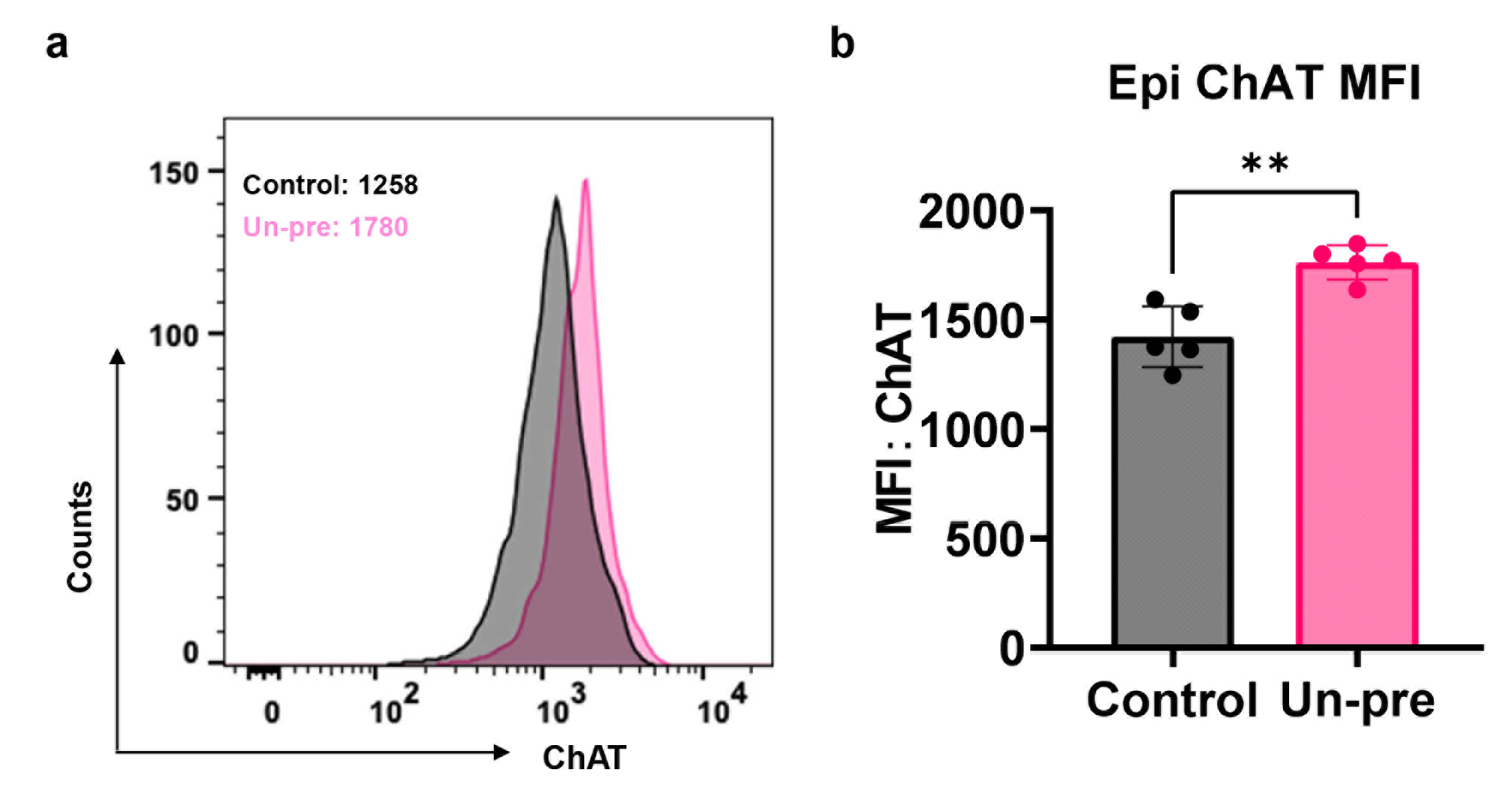

Supplement: Supplementary file 3 — Additional file 3: Supplementary Fig. S3. ChAT expression in pulmonary epithelial cells in model mice with chronic unpredictable stress. a. FACS analysis of ChAT expression in EpCAM+ epithelial cells in lungs of model mice with or without chronic unpredictable stress for 2 weeks. b. Mean fluorescence intensity of ChAT in pulmonary epithelial cells of model mice in the Control and Un-pre groups. **p < 0.01 [file 13046_2023_2836_MOESM3_ESM.png]

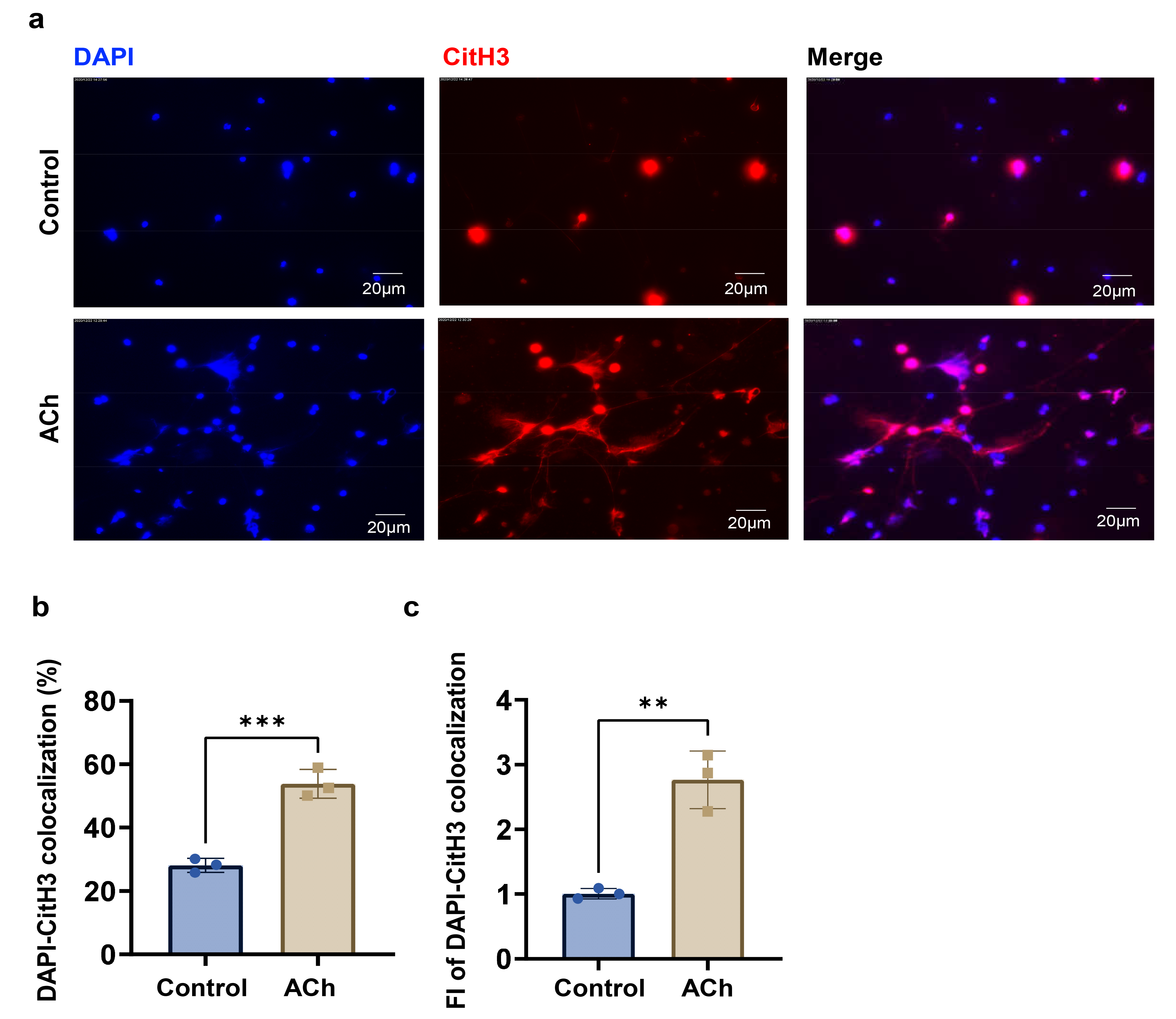

Supplement: Supplementary file 4 — Additional file 4: Supplementary Fig. S4. ACh promotes NETosis of neutrophils isolated from lungs of 4T1 orthotopic injection breast cancer model mice in the control group. a. 1 × 10^4^ neutrophils isolated from lungs of model mice in the control group at 1 week were treated with 100 μM ACh for 4 h. NETosis was examined by immunofluorescence. b-c. Percentage of NETosis (b) and fold change of fluorescence intensity of colocalization (c). **p < 0.01; ***p < 0.001 [file 13046_2023_2836_MOESM4_ESM.png]
