# Supplementary material for: Chronic stress induces pulmonary epithelial cells to produce acetylcholine that remodels lung pre-metastatic niche of breast cancer by enhancing NETosis
Source: J Exp Clin Cancer Res. 2023 Sep 29;42:255. doi: 10.1186/s13046-023-02836-5 (PMC10540414; doi:10.1186/s13046-023-02836-5)
Supplement: Supplementary file 5 — Additional file 5: Supplementary Table S1. Primers used in qRT-PCR [file 13046_2023_2836_MOESM5_ESM.docx]

**Supplementary Table S1.** Primers used in qRT-PCR

| Name | Sequences (5’→3’) | |
| --- | --- | --- |
|  | Forward | Reverse |
| *ChAT* | CCATTGTGAAGCGGTTTGGG | GCCAGGCGGTTGTTTAGATACA |
| *VAChT* | GCCCATTGTTCCCGACTATATC | AATAGCACGCCTATCTTCACATC |
| *PADI4* | TCTGCTCCTAAGGGCTACACA | GTCCAGAGGCCATTTGGAGG |
| *CXCR2* | ATGCCCTCTATTCTGCCAGAT | GTGCTCCGGTTGTATAAGATGAC |
| *β-Actin* | GGCTGTATTCCCCTCCATCG | CCAGTTGGTAACAATGCCATGT |
