# Supplementary material for: Chronic stress induces pulmonary epithelial cells to produce acetylcholine that remodels lung pre-metastatic niche of breast cancer by enhancing NETosis
Source: J Exp Clin Cancer Res. 2023 Sep 29;42:255. doi: 10.1186/s13046-023-02836-5 (PMC10540414; doi:10.1186/s13046-023-02836-5)
Supplement: Supplementary file 6 — Additional file 6: Supplementary Table S2. Clinicopathological characteristics of female breast cancer patients with lung metastasis [file 13046_2023_2836_MOESM6_ESM.docx]

**Supplementary Table S2. Clinicopathological characteristics of female breast cancer patients with lung metastasis**

| No | Age  (Year) | Metastasis (years  after diagnosis) | ER | PR | HER2 | Ki67 | | Histological classification | |
| --- | --- | --- | --- | --- | --- | --- | --- | --- | --- |
| 1 | 50 | 3 | － | － | ＋ | | 50% | | HER2 |
| 2 | 63 | 34 | ＋ | ＋ | ＋ | | 20-30% | | Luminal B |
| 3 | 43 | 2 | － | － | ＋ | | ＋ | | HER2 |
| 4 | 46 | 3 | ＋ | ＋ | － | | 2＋ | | Luminal A |
| 5 | 56 | 2 | ＋ | － | － | | 60% | | Luminal B |
| 6 | 50 | 4 | ＋ | － | － | | 90% | | Luminal B |
| 7 | 55 | 16 | ＋ | ＋ | － | | 60% | | Luminal B |
| 8 | 62 | 6 | ＋ | － | － | | 2% | | Luminal A |
| 9 | 54 | 1 | － | ＋ | ＋ | | 20% | | Luminal B |
| 10 | 48 | 14 | ＋ | ＋ | － | | 10% | | Luminal A |
| 11 | 48 | 1 | － | － | － | | 40% | | TNBC |
| 12 | 43 | 1 | － | － | － | | 70% | | TNBC |
| 13 | 63 | 13 | ＋ | － | － | | 60% | | Luminal B |

TNBC: triple negative breast cancer
