# Supplementary material for: Chronic stress induces pulmonary epithelial cells to produce acetylcholine that remodels lung pre-metastatic niche of breast cancer by enhancing NETosis
Source: J Exp Clin Cancer Res. 2023 Sep 29;42:255. doi: 10.1186/s13046-023-02836-5 (PMC10540414; doi:10.1186/s13046-023-02836-5)
Supplement: Supplementary file 7 — Additional file 7: Supplementary Table S3. Clinical information of non-tumor patients with pulmonary diseases [file 13046_2023_2836_MOESM7_ESM.docx]

**Supplementary Table S3**. **Clinical information of non-tumor patients with pulmonary diseases**

| No | Sex | Age (y) | Diagnosis |
| --- | --- | --- | --- |
| 1 | F | 40 | Pulmonary cryptococosis |
| 2 | F | 60 | Pulmonary cryptococosis |
| 3 | F | 20 | Pneumomycosis |
| 4 | F | 50 | Pulmonary cryptococosis |
| 5 | F | 57 | Pulmonary granuloma without fungus |
| 6 | F | 45 | Pulmonary granuloma without fungus |
| 7 | F | 74 | Pulmonary granuloma without fungus |
| 8 | F | 58 | Pulmonary granuloma without fungus |
| 9 | F | 55 | Pneumomycosis |
| 10 | F | 61 | Pulmonary granuloma without fungus |
| 11 | F | 35 | Gangrenous granuloma of lung |
| 12 | F | 42 | Pneumomycosis |
| 13 | F | 78 | Pulmonary granuloma without fungus |
| 14 | F | 33 | Pneumomycosis |
| 15 | F | 72 | Pulmonary cryptococosis |
